# Supplementary material for: Evaluation of intervention fidelity of a complex psychosocial intervention Lifestyle Matters: a randomised controlled trial
Source: BMJ Open. 2021 Apr 8;11(4):e043478. doi: 10.1136/bmjopen-2020-043478 (PMC8039267; doi:10.1136/bmjopen-2020-043478)
Supplement: Supplementary data [file bmjopen-2020-043478supp001.pdf]

Lifestyle Matters Additional File 1

Contents

Document 1: Lifestyle Matters training delivery observation checklist 2

Document 2: Lifestyle Matters intervention delivery video checklist 3

Document 1: Lifestyle Matters training delivery observation checklist

**TRAINER**	
**Domain**	**Criteria**
Trainer	1. The trainer is the same for all training
	2. The trainer is an author of the programme
	3. Demonstrates a range of group work skills (didactic teaching, peer sharing, interactive activities, active experimentation etc)
Materials	4. PowerPoint presentations and materials directly relate to the goals of the training
	5. Provides a CD of training content and materials to all trainees
Tailoring	6. Training is tailored to recipients' skill level, experience or work background
	7. Training is tailored in response to the observed needs of the trainees, and feedback from the trainees during the sessions
Content	8. The goals of the training are covered
	9. Examples of the core themes and examples of subsidiary themes are covered
	10. Mandatory themes are covered:<br>• A celebration of achievements<br>• Activity and health<br>• Endings
**TRAINEES**	(Performance in discussion, role play, problem solving, expressive exercises, reflective exercises shared, written tests)
**Domain**	**Criteria**
Understanding	11. Critically discuss demographic changes and the concept of active ageing
	12. Discuss the evidence for Lifestyle matters
	13. Identify the ideas that are key to Lifestyle Matters, including<br>• enabling group members to do things for themselves<br>• transferring skills from the safety of the group to their wider lives
	14. Discuss the content of the Lifestyle Matters manual and how to apply it
Self -awareness	15. Share their prior expertise, hopes and expectations of the training
	16. Share their own perceptions of ageing and how this may impact on the programme
	17. Reflect on and share their own facilitation style and skills
Skill acquisition	18. Use the LifeStyle Matters manual to practice planning sessions within themes
	19. Identify how to locate and access community resources
	20. Respond to potential scenarios, using creative problem solving and considering individual needs (including risk taking)
Group work	21. Understand and demonstrate a range of group work skills (didactic teaching, peer sharing, interactive activities, active experimentation etc)
	22. Understand and demonstrate how to manage group dynamics
	23. Understand and demonstrate an enabling style of group facilitation
	24. Demonstrate how to tailor the intervention to the needs and choices of group members
Individual sessions	25. Explore the value of 1:1 sessions and potential activities to use in these
Group outings	26. Explore the value or outings and how to help group members to plan them
Reflection & evaluation	27. Explore reflective practice and discuss its value
	28. Practice evaluating and planning actions
Supervision	29. Discuss the value and principles of supervision

Document 2: Lifestyle Matters intervention delivery video checklist

**FACILITATOR**	
**Domain**	**Criteria**
Resources	30. Two facilitators deliver
Personal qualities	31. Shows warmth and empathy
	32. Listens actively and respectfully
Enabling	33. builds on the assets, skills and abilities that each person brings
	34. Encourages the group to make choices
Group work skills	35. Facilitates communication and interaction (to ground rules?)
	36. Draws in quiet members of the group
	37. Facilitates peer exchange and guidance, and peer modelling
	38. Manages space, time and opportunity effectively
	39. Facilitates the members to explore differences between them, or conflict and resolution
	40. Builds mutual support and group cohesion
Goals and needs	41. Balances meeting individual needs and goals and group needs
	42. Balances sufficient challenge in activities with ensuring they are achievable
	43. Tailors activities to the needs of members
Content	44. Focuses on the themes in the programme, or new themes chosen by group members
	45. Uses group activities, not just discussion (activities from the manual or from elsewhere)
	46. With members, selects and plans group outings that explore and extend the themes in the programme
**GROUP MEMBERS**	
**Domain**	**Criteria**
Understanding	47. The relationship between activity, health and aging, or one of the other themes from the programme
Occupational self-analysis	48. Analyse their own daily occupations (their significance, impact on health, components) and make choices
	49. Sharing and reflecting on experiences of participating in activities (Occupational storytelling)
Goal setting	50. Own goals identified within the group and within activities
Grading and layering	51. Grading and layering of activities to constantly revisit and extend skills and build confidence
Mutual support	52. Show mutual appreciation and support
Creativity	53. Practice any of: self-expression, innovation, problem solving and adaptation within the safety of the group
	54. Engage in enjoyable and stimulating activities
Older people as experts	55. Acknowledge and appreciate their own skills and how they can contribute to the group
	56. Bring in and share their own skills and knowledge
	57. Group members in turn take leadership or contribute ideas
Transfer to everyday life	58. build skills, solutions or confidence within the group
	59. Report the transferring of skills, solutions or confidence from the group to wider life
Change over time	60. Plans for individuals are taken forward, whilst respecting each person's need to balance opportunities and risk
Confidence and hope	61. Achievements are celebrated
	62. Adventures and dreams are welcomed
