# Supplementary material for: Evaluation of intervention fidelity of a complex psychosocial intervention Lifestyle Matters: a randomised controlled trial
Source: BMJ Open. 2021 Apr 8;11(4):e043478. doi: 10.1136/bmjopen-2020-043478 (PMC8039267; doi:10.1136/bmjopen-2020-043478)
Supplement: Author's manuscript [file bmjopen-2020-043478.draft_revisions.pdf]

BMJ Open is committed to open peer review. As part of this commitment we make the peer review history of every article we publish publicly available.

When an article is published we post the peer reviewers' comments and the authors' responses online. We also post the versions of the paper that were used during peer review. These are the versions that the peer review comments apply to.

The versions of the paper that follow are the versions that were submitted during the peer review process. They are not the versions of record or the final published versions. They should not be cited or distributed as the published version of this manuscript.

BMJ Open is an open access journal and the full, final, typeset and author-corrected version of record of the manuscript is available on our site with no access controls, subscription charges or pay-per-view fees (http://bmjopen.bmj.com).

If you have any questions on BMJ Open's open peer review process please email info.bmjopen@bmj.com

BMJ Open

Evaluation of intervention fidelity in a randomised controlled trial of a complex psychosocial intervention: Lifestyle Matters

Journal:	*BMJ Open*
Manuscript ID	bmjopen-2020-043478
Article Type:	Original research
Date Submitted by the Author:	04-Aug-2020
Complete List of Authors:	Sprange, Kirsty; The University of Nottingham, Nottingham Clinical Trials Research Unit, University Park, NG7 2RD<br>Mountain, Gail; University of Bradford, Centre for Applied Dementia Studies, Faculty of Health Studies, Richmond Road, Bradford, BD7 1DP<br>Craig, Claire; Sheffield Hallam University, Lab4Living, Furnival Building, 153 Arundel Street, Sheffield, S1 2NU
Keywords:	Dementia < NEUROLOGY, GERIATRIC MEDICINE, Clinical trials < THERAPEUTICS

SCHOLARONE™
Manuscripts

I, the Submitting Author has the right to grant and does grant on behalf of all authors of the Work (as defined in the below author licence), an exclusive licence and/or a non-exclusive licence for contributions from authors who are: i) UK Crown employees; ii) where BMJ has agreed a CC-BY licence shall apply, and/or iii) in accordance with the terms applicable for US Federal Government officers or employees acting as part of their official duties; on a worldwide, perpetual, irrevocable, royalty-free basis to BMJ Publishing Group Ltd ("BMJ") its licensees and where the relevant Journal is co-owned by BMJ to the co-owners of the Journal, to publish the Work in this journal and any other BMJ products and to exploit all rights, as set out in our licence.

The Submitting Author accepts and understands that any supply made under these terms is made by BMJ to the Submitting Author unless you are acting as an employee on behalf of your employer or a postgraduate student of an affiliated institution which is paying any applicable article publishing charge ("APC") for Open Access articles. Where the Submitting Author wishes to make the Work available on an Open Access basis (and intends to pay the relevant APC), the terms of reuse of such Open Access shall be governed by a Creative Commons licence – details of these licences and which Creative Commons licence will apply to this Work are set out in our licence referred to above.

Other than as permitted in any relevant BMJ Author's Self Archiving Policies, I confirm this Work has not been accepted for publication elsewhere, is not being considered for publication elsewhere and does not duplicate material already published. I confirm all authors consent to publication of this Work and authorise the granting of this licence.

For peer review only

Evaluation of intervention fidelity in a randomised controlled trial of a complex psychosocial intervention: Lifestyle Matters

Authors

Kirsty Sprange, University of Nottingham, Nottingham Clinical Trials Unit, University Park, NG7 2RD, UK. kirsty.sprange@nottingham.ac.uk (Corresponding author) ORCiD 0000-0001-6443-7242

Gail A Mountain University of Bradford, Centre for Applied Dementia Research, Faculty of Health Studies, Richmond Road, Bradford, BD7 1DP, UK g.mountain@bradford.ac.uk ORCiD 0000-0002-5417-7691

Claire Craig, Sheffield Hallam University, Lab4Living, Furnival Building, 153 Arundel Street, Sheffield, S1 2NU, c.craig@shu.ac.uk ORCiD 0000-0002-3475-3292

For peer review only

Abstract

Objectives: Robust research of complex interventions designed to promote mental wellbeing in later life is required to inform service development. An essential component is ensuring that such interventions are delivered as intended. This paper presents a detailed description of the design and implementation of a fidelity assessment within a trial of one such intervention (Lifestyle Matters). The findings help to explain the trial results and also inform the design of embedded fidelity assessments within future evaluations of complex interventions.

Design: We conducted a comprehensive mixed method fidelity assessment embedded as part of a multi-centre pragmatic, parallel group, individually randomised controlled trial. A conceptual fidelity framework was developed, underpinned by mechanisms to facilitate behaviour change and from this the fidelity assessment was designed. The resulting instrument assessed the following parameters: intervention design, training and supervision; and delivery, receipt and enactment of the intervention.

Intervention: The Lifestyle Matters intervention uses an occupational approach to healthy ageing with the aim of improving and sustaining metal wellbeing. It incorporates a mix of facilitated group meetings and one-to-one sessions to enable participants to redesign their lifestyle.

Results: The fidelity assessment demonstrated that the intervention was delivered as per protocol and was tailored to meet individual needs. There was substantial inter-rater agreement for training and group member performance 0.72; and moderate inter-rater agreement for facilitator performance 0.55. It was not possible to determine whether small declines seen in facilitator performance were due to facilitator drift or moderating factors such as group dynamics or participant characteristics.

Conclusions: The assessment methods used adequately measured criteria identified as being significant indicators of fidelity. Adherence during training, delivery and supervision was good. The subjective nature of identification and rating observed behaviours was the main challenge. Future research should explore alternative methods of assessing fidelity in trials of complex interventions.

For peer review only

Trial registration: ISRCTN67209155

Keywords: Fidelity assessment, complex intervention, dementia

Strengths and Limitations of this study

- This study demonstrated feasibility to design a fidelity assessment framework and develop bespoke assessment tools to evaluate a complex multi-component psychosocial intervention.
- The extent of the assessment was limited by the methods used, for example it was not appropriate or even ethical to undertake observations of activities in the community.
- The validity of the findings is reliant on whether bespoke assessment tools are sensitive enough to identify the complex constructs within this type of intervention.

Background

An increase in long term conditions within the global population combined with increased longevity has led to interest in how tailored psychosocial interventions might enable individuals to retain physical and mental wellbeing in the extended lifespan. UK guidance has identified the need for multi-component, tailored individual and group based preventative interventions which promote health and wellbeing through occupation and physical activity [1]. The benefits of social networking and group interventions involving activity targeting social isolation and loneliness has also been identified [2] but there is a limited range of recommended interventions. The challenges of associated research include intervention development and evaluation of intervention acceptability, followed by robust testing to determine intervention effectiveness [1]. Robust testing should include an assessment of intervention fidelity to assess the extent to which the intervention is delivered as intended.

Fidelity assessment maximises validity, reliability, effect size and improves statistical power, [3-5] enabling researchers to demonstrate with greater confidence that study results were due to the intervention and not to other confounding factors. Additionally observing causal mechanisms, how they produce change in a particular setting and how this context affects implementation is essential for effective intervention implementation [6] and for promulgation of evidence based practice [7]. It is acknowledged that using multiple fidelity assessment procedures can enhance validity and reliability of complex interventions[5].

For peer review only - http://bmjopen.bmj.com/site/about/guidelines.xhtml

A pragmatic randomised controlled trial (RCT) with an embedded fidelity assessment was conducted to evaluate a complex manualised intervention, Lifestyle Matters, which was created to promote the mental wellbeing of community living older people [8]. Lifestyle Matters was developed for the UK setting from a US intervention, Lifestyle Redesign which was found to promote physical and mental health and wellbeing, occupational functioning and life satisfaction in older adults [9 10]. As reported in [8], the trial fully adhered to Consort guidelines.

Fidelity assessment for the Lifestyle Matters study included an evaluation of facilitator training and intervention delivery against pre-determined criteria; and a qualitative exploration of the mechanisms that mediated or moderated lifestyle change in study participants; thereby identifying causal mechanisms associated with implementation.

There is some debate about the benefits or otherwise of the manualisation of complex interventions. It has been observed that manaualisation can reduce variance in intervention delivery thereby enhancing fidelity [11]. Alternatively it can promote a generic approach which stifles flexibility, inhibits facilitator creativity and ignores the individual needs of participants [7 12]. As described below, Lifestyle Matters is underpinned by core principles but also promotes participant choice.

MRC guidance recommends that complex intervention design and the associated fidelity assessment frameworks are developed from the underlying intervention theory [13]. Lifestyle Matters is underpinned by social cognitive theory. There is a focus upon promotion of self-management by participants [14], and in particular upon developing and sustaining self-efficacy as this is known to be a protective factor in improving motivation and emotional wellbeing [15].

Interventions like Lifestyle Matters will have core components such as number of group and individual sessions and structured manual topics. However adapting a complex intervention to meet the needs of individual participants is important [16 17]. The Lifestyle Matters intervention provides a content and delivery structure, which is both flexible and readily tailored to the needs of participants. This necessitated the application of a range of methods and tools to assess and evaluate fidelity.

For peer review only - http://bmjopen.bmj.com/site/about/guidelines.xhtml

This paper describes the fidelity assessment conducted for the Lifestyle Matters study and presents the findings from analysis of facilitator training and supervision, intervention delivery and receipt. The findings are then discussed in the context of intervention implementation.

Trial design

The study was a pragmatic, two-arm, parallel group, individually randomised controlled trial of 288 participants, designed to determine the population benefit of an occupation based intervention for people aged 65 years or older [8]. 288 participants were randomised between 14 August 2012 and 19 April 2013 (18 couples and 252 individuals); 145 and 143 were allocated to the intervention and control groups, respectively. The intervention was delivered in one city in the north of England and in North Wales. Participants were eligible if they had reasonable cognitive function to participate in the intervention as evidenced by a score of 0 to 7 on the Six Item Cognitive Impairment Test (6CIT). They were also living independently or in sheltered accommodation, alone or with others and able to converse in English or Welsh. An associated fidelity assessment and qualitative sub-study were conducted to evaluate facilitator training and supervision; intervention delivery; and explore mechanisms that mediate or moderate lifestyle change by participants.

Patient and public involvement

Patients and members of the public were involved at all stages from the development of the intervention during feasibility testing through to trial design and conduct. We received input from older adults on the design of the trial materials and in the oversight of the delivery of the study through PPI representation on trial oversight groups. The main trial results were disseminated to participants using materials suitable for a non-specialist audience and shared with the wider public, clinical and academic colleagues through events and publications.

The Lifestyle Matters intervention

The intervention involves volunteers taking part in sixteen facilitated sequential weekly group meetings with up to 15 others and in four one-to-one sessions with one of the facilitators to pursue individual goals [8]. During group meetings participants are encouraged to think about and engage in discussion and activities to explore the relationship between occupation and health, selecting from a menu of potential topics. A crucial element is going

For peer review only - http://bmjopen.bmj.com/site/about/guidelines.xhtml

into the community to try out new or neglected skills with support from each other and from the facilitators; at least four sessions should involve such activities.

Group meetings are approximately two hours in duration at a convenient community venue. The first group sessions are structured to cover key topics from the manualised intervention. This provides a foundation for facilitators to maintain adherence to the intervention and for both facilitators and participants to work together to on future sessions using the same model. Short breaks of no more than two weeks can be included to accommodate statutory holidays.

One-to-one sessions are an important intervention component and are specifically directed by the individual's goals and preferences. The first session concentrates on the participant-facilitator relationship. The remaining three sessions are built around the participant and what they want to achieve, using the intervention to assist them to execute this.

Facilitators and facilitation

Facilitators with the necessary skill set and qualities were recruited to deliver the intervention as intended [12]. They had to have prior experience of working with groups and with older people. A requirement of intervention delivery is for two facilitators to be present during each group and to share working with participants during one-to-one sessions.

Facilitator Supervision

Supervision was provided by appropriately experienced Occupational Therapists who also attended facilitator training alongside the facilitators. A protocol was created to enable provision of consistent and appropriate supervision across and within sites, initially on a one to one basis and later to include teleconferencing or joint supervision with other facilitators if appropriate.

Training

A two day intensive training course was delivered jointly to all facilitators and supervisors before intervention delivery commenced, thereby ensuring that everyone received the same information and training experience to equip them to deliver the intervention and improve fidelity [18].

For peer review only

Training content included presenting the rationale and theory behind the intervention and intervention components [12]. The training then mirrored delivery of Lifestyle Matters intervention and manual in that it involved;

- understanding the importance of taking a personalised and tailored approach towards participants at all times [19].
- didactic sessions to explain the programme and its ethos.
- peer sharing; providing the opportunity to share narratives, explore experience and attitudes and reflect on the relationship that can develop between participants and between facilitators and participants and between supervisor and facilitators.
- undertaking active experimentation, thereby testing out ideas and concepts.
- exploring communication skills through role playing, vignettes and group discussions.

Part of the two day training was video recorded to give facilitators an idea of how they and their participants might experience this for fidelity assessment and smooth out any challenges with using the recording equipment.

All facilitators and supervisors received a copy of the manual and an accompanying CD-Rom with downloadable resources for intervention delivery [20].

Materials and methods

Fidelity assessment framework

A comprehensive fidelity assessment framework specific to the intervention was developed (see Table 1) based on the Behaviour Change Consortium (BCC) recommendations and NICE guidance on behaviour change [3 21]. Fidelity assessment and quality assurance parameters centred on the BCC five recommended criteria: intervention design, training, delivery, receipt and enactment. For each criterion, programme objectives based on the Lifestyle Matters manualised intervention and training were discussed and agreed by two senior researchers. A description for each standard was then developed and methods to demonstrate fidelity to each standard identified (see Table 1).

[TABLE 1]

For peer review only - http://bmjopen.bmj.com/site/about/guidelines.xhtml

Assessment tools

The underlying ethos of Lifestyle Matters and its tailored approach to delivery make fidelity assessment complex as many of the identified criteria are subjective. A range of quantitative and qualitative tools were therefore used in line with MRC recommendations for complex interventions [17], including attendance registers, observation checklists and interview schedules which were all piloted before application.

Attendance registers

Registers were used to record both group and individual session attendance. Using pilot study findings It was estimated that participants would need to attend at least half (8) group meetings, to benefit from the intervention [22]. The number of individual sessions required to derive benefit was not set.

Development of observation checklists

Observation checklists developed by two senior researchers identified a number of domains comprising the core skills and key criteria identified in the Lifestyle Matters manual, worksheets and training materials, see additional files 1 and 2. Criteria described components and behaviours expected to be present during intervention delivery. Domains were divided into those associated with 'Trainer or Trainee performance' and those associated with 'Facilitator or Group member performance'.

A scoring system was devised. A number of criterion were rated on a binary 'Yes' or 'No' scale for presence or absence. For the purpose of analysis if the criterion was present this was converted to a score of 3. If the criterion was not present this was converted to a score of 0.

Fidelity scores were calculated based on agreement of a final score between the two coders and the total was given a percentage score as follows:

- 0- 60% Unsatisfactory
- 61-70% Satisfactory
- 71-80% Good
- 81-90% Very good
- 91-100% Excellent

For peer review only - http://bmjopen.bmj.com/site/about/guidelines.xhtml

To ensure consistency the same two researchers completed fidelity assessment activities and coding. The patient and public contributor for the project also contributed to the fidelity assessment analysis including reviewing and piloting observation checklists (as described below), as well as participant observation during training.

Assessment of the fidelity of training delivery

Participant observation, in which the researcher not only observes, but takes an active role in the setting [23], was selected to assess the fidelity of training delivery. The researchers therefore attended the training as delegates. During the training, sessions were independently rated by two researchers. There were also discussions between the trainer and researchers at the beginning and end of each day to review content and feedback from attendees. All scores were then shared and reviewed for reliability of interpretation and coding against checklist criteria.

Assessment of the fidelity of intervention delivery

A purposive sample of eight video-recordings were taken of four of the 11 Lifestyle Matters groups to monitor implementation and adherence to the intervention and identify any facilitator drift over time. Groups from both sites were selected for assessment. Written consent to conduct video recordings was obtained from each participant and facilitators recorded the group meetings using simple compact video recorders.

One video of each of the four selected groups was recorded at approximately week four and again at around week 10 of intervention delivery although some flexibility in session choice was necessary to avoid out of venue activities (see Table 2).

[TABLE 2]

Video content was then rated using the predetermined checklist and findings analysed to determine delivery and receipt of the intervention and any change over time.

During piloting of the intervention fidelity checklist it became apparent that several of the assessment criteria could not be observed during the weekly group meetings. For example receipt and enactment of the intervention would occur in the participant's everyday life away from the group. It was deemed that either the criterion could not be present during every

For peer review only - http://bmjopen.bmj.com/site/about/guidelines.xhtml

weekly meeting or it would be necessary to observe several meetings consecutively which was not planned. Further observations using the checklist to test the assumption found this to be consistent. The criteria deemed to be immeasurable were therefore excluded from the final analysis.

Due to practicalities, only the groups held within the meeting venue were observed. Given the importance of enactment of activities in the community this was a limitation.

Inter-rater reliability

Inter-rater reliability demonstrates whether available data accurately represents the variables being measured and the extent to which coders attribute the same score to the same variable [24]. Inter-rater reliability was calculated by two researchers independently coding video recordings and by scores being compared using the Kappa statistic, with a value of 0.61 and above indicating good inter-rater agreement.

- < 0.20 Poor
- 0.21-0.40 Fair
- 0.41-0.60 Moderate
- 0.61-0.80 Good
- 0.81-1.00 Very good

Semi-structured interviews

Semi-structured interviews were undertaken with all facilitators to explore views on what enables effective intervention facilitation, and the skills and competencies required to deliver the intervention and enable participants to act on it [8]. Interviews took place at the end of the first and third (final) wave of intervention delivery to identify any changes in experiences of delivery over time.

A purposeful sample of 10% of participants were interviewed; selected from both sites and each of three waves of intervention delivery to elicit the range and nature of issues that influenced their experiences of the intervention.

All transcripts were read for familiarisation and annotated for themes by the same two researchers using Framework analysis in NVivo [25 26]. For the purposes of the fidelity

For peer review only - http://bmjopen.bmj.com/site/about/guidelines.xhtml

assessment, data from the interviews to identify cross cutting themes and explanatory factors for fidelity outcomes.

Results

The 'Fidelity Assessment Framework' including evidence for fidelity is presented in table 1.

Facilitators

All four facilitators remained the same throughout the trial at both sites. They were all female, younger than the youngest participant (65 years) and from a health or social care professional background.

Attendance

Eleven Lifestyle Matters interventions were delivered, six in one city in Northern England and five in North Wales. Four groups were selected for the fidelity assessment as previously described. Attendance at each was 70% or above (see table 2). The average number of out of venue activities conducted as a part of the group aspect of the intervention across all groups was four.

Training delivery

Excellent fidelity was demonstrated for trainer performance with a score of 100% across all four domains (see Table 3).

[TABLE 3]

Good fidelity was identified through a criterion score of 75.4% across eight domains for trainees. Overall training fidelity was very good with a total agreed criterion score for 'trainer performance' and 'trainee performance' of 83.9%.

Intervention delivery

Facilitator performance

Excellent or very good fidelity was achieved in four out of the eight recorded groups (see Table 4). Only one observed group (Wave 1, Site A) demonstrated unsatisfactory fidelity (52%).

For peer review only - http://bmjopen.bmj.com/site/about/guidelines.xhtml

[TABLE 4]

Excellent or very good fidelity was demonstrated in three of the six assessment domains. Only one domain; 'Goals and Needs' was rated as being unsatisfactory. This required evidence of programme tailoring and how facilitators helped participants to select achievable activities with appropriate levels of challenge.

The decline in fidelity seen at Site A during Wave 1 (77% to 52%), could be explained by the facilitators arranging for two external speakers to deliver sessions on relaxation and physical health. During the meeting the facilitators largely left facilitation to the external speakers who used a didactic style, which did not invite discussion or tailoring to the needs of group members. It was deemed by both coders that the facilitators could have coached external speakers on how to deliver a session in accordance with the Lifestyle Matters ethos, and could have interjected during the session. An outcome from this particular session was to provide facilitators with further guidance on when and how to engage external speakers. This was delivered as an email to all facilitators ensuring they received consistent information.

A difference was also noted between the first and last waves at Site B where the assessed fidelity dropped from 87.5% to 70.8% but was still within the boundary set for satisfactory fidelity. Based on findings from the qualitative interviews, reasons for the reduction in scores could be explained by difficulties in time management to undertake planning and delivery of multiple consecutive groups.

Group member performance

Excellent or very good fidelity was demonstrated for four out of the eight recorded groups (see Table 5). Only one group (Wave 1, Site A) was rated as being unsatisfactory (41%) which was related to the same issue of 'Facilitator performance'.

[TABLE 5]

Excellent or very good fidelity was demonstrated for three out of eight identified domains with unsatisfactory evidence for only one; 'Confidence and hope'. However it was evident that ability to score some criteria was affected by the nature of the activities participants engaged in.

For peer review only - http://bmjopen.bmj.com/site/about/guidelines.xhtml

Inter-rater reliability

Kappa scores were calculated using linear weights [27].

For the assessment of training fidelity the inter-rater reliability between coders was Kappa = 0.72 (p <.0.001), 95% CI (0.478, 0.964).

Inter-rater reliability for overall fidelity to intervention delivery across sites was Kappa = 0.63 (p <0.001), 95% CI (0.543, 0.719).

Inter-rater reliability for facilitator domains across both sites was Kappa = 0.55 (p <0.001), 95% CI (0.432, 0.678). For group members inter-rater reliability was Kappa = 0.72 (p <0.001), 95% CI (0.598, 0.844).

Qualitative findings

Post group delivery Facilitator interviews

Analysis of facilitator interviews revealed that they all considered that they had become more confident, skilled and flexible over time. Also that their commitment to the ethos of the programme was maintained:

> "*We've actually grown in confidence…it feels a very different place to where we were at the beginning... far more skilled... allowing for that flexibility and spontaneity" (Facilitator).*

Further training opportunities such as booster sessions or shadowing experienced facilitators were suggested to help consolidate knowledge of the intervention and improve fidelity further.

> *"…it would have been useful just to have…a follow up session just to see what kinds of things were coming up for people and have that, that kind of discussion" (Facilitator).*

Regular self-review, using a group observation checklist was proposed by facilitators as a tool to enable them to reflect on delivery style and decision-making processes. (Facilitators were aware of the checklist as part of the fidelity work, but did not have sight of the checklist).

For peer review only - http://bmjopen.bmj.com/site/about/guidelines.xhtml

"it may be more useful in future, if the facilitators could peer review themselves every three weeks against the checklist…scoring themselves, and detailing an action against those that need to be achieved" (Facilitator).

Two facilitators reported levels of performance anxiety due to the session being recorded, which they felt may have hindered their delivery of the intervention

"I think it is fair to say that being recorded has significantly affected the performance of the facilitators due to nerves of being recorded. As such I don't think the recordings reflect what has actually taken place within the groups over time. We tick many of the boxes on the checklist, but the recordings I feel won't pick a lot of this up" (Facilitator).

Discussion

The fidelity assessment framework designed for this study provided a satisfactory structure for monitoring and evaluating adherence to the programme over time. However frameworks are designed to look for a list of set criteria, which by definition should be the same for all observations. This is not necessarily true of complex interventions such as Lifestyle Matters that have customisation as an underlying mechanism. For example we expected to observe group and individual goal setting opportunities in intervention sessions. However the majority of participants struggled to engage with the concept of 'goal setting' and facilitators were required to make significant changes to their own understanding of 'goal setting' and how they presented the concept to participants. Low fidelity therefore may not necessarily be associated with poor fidelity to an intervention but may highlight a problem with the original intervention presumptions, nature of associated training and supervision, and subsequent criteria identified to evidence fidelity. Detailed analysis of feasibility/pilot findings is therefore essential to establish clear baselines for behavioural interventions. Good design and piloting of tools is also important.

Coding

Coding performance can raise concerns. Expectations of both the facilitators and those rating performance must be considered and all involved should try to avoid pre-occupation with fidelity assessment at the cost of intervention delivery[7]. Coders were not independent of the study, having attended the training as participant observers. Using independent coders would reduce bias of unknown factors arising from personal knowledge of facilitators and increase

For peer review only - http://bmjopen.bmj.com/site/about/guidelines.xhtml

the rigour of fidelity assessment. However when the intervention is complex, ability to be able to interpret what is occurring is required if accurate observations are to be recorded[28].

Training

One of the main challenges to fidelity was that of delivering high quality in-depth training for a complex psycho-social intervention in just two days to a range of staff. Chances of variation in intervention delivery were deemed to lie in facilitator background and experience which are related to fidelity [5]. The Lifestyle Matters manual and training are founded on 'occupational therapy' (OT) theory and practice and the facilitators were supervised by trained OTs. However in pursuance of evidence that the intervention might be delivered by a range of staff, the recruited facilitators came from a variety of professional backgrounds including social work and mental health advocacy as well occupational therapy. Further customisation of training may be required or a range of training programmes devised a wider range of professional and non-professional staff.

Some deterioration of skills, or drift, is common post training [12]. Although no additional training or booster sessions were provided during this study, facilitators received regular supervision and occasionally received emailed 'study updates'. These communications (approved by the Trial Management Group), clarified details of the programme to help reduce potential facilitator drift. Any ethical concerns arising would have been acted on if identified. Although fidelity was generally found to be good, further training opportunities such as booster sessions or shadowing experienced facilitators might be incorporated in further studies to test whether additional training consolidates knowledge of the intervention and further improves fidelity.

Facilitators and supervisors

It has been suggested that due to the key role of interventionists they should be cognisant of the impact of fidelity [5]. The importance and nature of the planned fidelity assessment was explained to the facilitators during the two day preparatory training before they took part in the assessment. Obtaining sign-up to the fidelity process at such an early stage was beneficial in providing reassurance about the observation processes.

Even though facilitators were from a range of backgrounds and had different experiences of working with older people in the community, this did not appear to negatively impact on

For peer review only - http://bmjopen.bmj.com/site/about/guidelines.xhtml

intervention delivery [29]. The facilitator job description and person specification provided a priori level of credentials required to provide some consistency across facilitators. However the previous point about responsiveness to needs for training and support should be maintained. This also applies to supervisors who are not experienced in delivering the programme.

A ‘Good fit’ between providers and participants can be key to successful intervention delivery [12]. All facilitators in this trial were female and younger than the youngest participant but this did not appear to impact on delivery. The supervisors were generally older and more experienced in delivering occupational therapy based interventions, which could be drawn upon during supervision sessions.

Receipt of intervention

Fidelity to the intervention increased between waves for Site A, but gradually decreased for Site B. This could have been due to the dynamics of the groups that were recorded; challenges with the methods used to assess fidelity, or may have been indicative of facilitator drift over time. The only factor that could be identified to explain facilitator drift at site B was the time pressure they expressed in trying to prepare and deliver the group sessions as well as trying to organise and conduct individual sessions. This explanation is commensurate with the slight decline seen in ‘Facilitator performance’ and suggests that additional training may have been beneficial.

Practicalities

The assessment only involved the video recording of a small number of weekly meetings out of the total provided and therefore it is inevitable that some facilitator and participant behaviours that may have affected intervention delivery were not observed. The assessment process also did not include observations of facilitators arranging and conducting individual sessions or out of venue activities which would have enhanced our understanding of the underlying intervention mechanisms.

Consideration should be given to how fidelity assessment can be undertaken within any given setting without interfering with the intervention [30]. How many, when and what type of sessions to record should be dependent on the complexity of the trial and availability of resources. For example it may be prudent to monitor consecutive sessions in order to capture

For peer review only - http://bmjopen.bmj.com/site/about/guidelines.xhtml

more information regarding participant receipt and enactment. Behaviour change may be more evident if an activity undertaken during one session can then be followed-through to the next session.

Pscychosocial interventions are not designed to be passive; participants and facilitators bring their life experience, beliefs and expectations [29 31]. When determining intervention fidelity it is therefore important to consider how group dynamics and also environmental factors [32] impact not only on group composition but also group behaviour, especially when external factors to the intervention may be outside the control of those facilitating [31].

Conclusions

The outcomes from this fidelity assessment add to the growing literature on the use of fidelity assessment in trials and other studies of complex interventions. Psychosocial interventions that are flexible and offer a tailored approach in delivery may make fidelity assessment difficult, but not impossible. Paradoxically the importance of undertaking thorough fidelity assessment is enhanced for complex interventions, which have a number of components. Despite the challenges, this study has demonstrated that by testing methods identified in a replicable framework, fidelity assessment can be carried out successfully.

Further research should explore the potential of different observational methods for monitoring fidelity in psycho-social community based complex trials and the impact of these methods on intervention delivery.

Abbreviations

Behaviour Change Consortium (BCC)

Randomized controlled trial (RCT)

Declarations

Ethics approval and consent to participate

The study was approved by the South Yorkshire Research Ethics Committee 12/YH/0101, the National Institute for Social Care and Health Research (NISCHR) Permissions Co-ordinating Unit in Wales, Betsi Cadwaladr University Health Board. Written informed consent was obtained from all participants.

For peer review only

For peer review only - http://bmjopen.bmj.com/site/about/guidelines.xhtml

Consent for publication

We obtained written informed consent for the participants who took part in the interviews via the Trial Consent Form under the following statement:

"I understand and give permission for group meetings or interviews that I may be asked to take part to be video/audio recorded and for the resulting data to be used in reports and publications including direct quotation."

This information is held as part of the archived record of the trial. Only anonymised non-identifiable data are used in this report as per written consent.

Availability of data and material

The datasets generated and analysed for this study will be available upon request from the authors.

Competing interests

GM and CC are the original authors of the published Lifestyle Matters manualised programme. No other authors have any competing interests.

Funding

The trial was funded through the Medical Research Council (MRC) Lifelong Health and Wellbeing (LLHW) programme, grant number G1001406, ISRCTN is 67209155. The views and opinions expressed in this paper are those of the authors and do not necessarily reflect those of the MRC. Primary Care Research Network (PCRN) funding was accessed to support recruitment activity in GP surgeries in Sheffield and NISCHR provided support in North Wales. The funder has reviewed and approved the trial design including methods of data collection, analysis and interpretation of data.

Author contributions

KS: Process evaluation and fidelity assessment design, development of framework, development and preparation of tools, data collection and analysis, development of manuscript. GM: Process evaluation and fidelity assessment design, development of framework, development of manuscript. CC: Delivery of training and development of training checklist. All authors have read and approved the manuscript.

For peer review only

Acknowledgements

PPI contributors. Prof John Brazier, Dr Daniel Hind, Prof Stephen Walters, Dr Gill Windle, Prof Robert Woods (TMG) advised on the trial protocol including the process evaluation and fidelity assessment. Dr Pip Logan, Dr Jennifer Wenborn, Dr Linda Sheppard, Dr Fiona Goudie of the Trial Steering Committee (TSC) and Dr Mona Kanaan, Prof Avril Drummond and Dr Claire Ballinger of the Data Monitoring and Ethics Committee (DMEC) advised on and critically reviewed the trial protocol including the process evaluation and fidelity assessment.

For peer review only

References

1. National Institute for Health and Care Excellence. Public health guideline [PH16]. Mental wellbeing in over 65s: occupational therapy and physical activity interventions 2008 [Available from: https://www.nice.org.uk/guidance/ph16 accessed 03Januray2018.

2. Government Office for Science. Foresight Mental Capital and Wellbeing: Making the most of ourselves in the 21st century 2008 [Available from: http://www.bis.gov.uk/assets/biscore/corporate/migratedD/ec_group/116-08-FO_b accessed January 2013.

3. Bellg A, Resnick B, Sharp Minicucci D, et al. Enhancing treatment fidelity in health behaviour change studies: Best practices and recommendations from the NIH Behaviour Change Consortium. *Health Psychology* 2004;23(5):443-51.

4. Resnick B, Bellg A, Borrelli B, et al. Examples of implementation and evaluation of treatment fidelity in the BCC studies: Where we are and where we need to go. *Annals of Behavioural Medicine* 2005;29(Special Supplement):46-54.

5. Spillane V, Byrne M, Byrne M, et al. Monitoring treatment fidelity in a randomised controlled trial of a complex intervention. *Journal of Advanced Nursing* 2007;60(3):343-52.

6. Craig P, Dieppe P, Macintyre S, et al. Developing and evaluating complex interventions: the new Medical Research Council guidance. *Bmj* 2008;337:a1655. doi: 10.1136/bmj.a1655 [published Online First: 2008/10/01]

For peer review only - http://bmjopen.bmj.com/site/about/guidelines.xhtml

7. Dumas J, Lynch A, Laughlin J, et al. Promoting Intervention Fidelity. Conceptual Issues, Methods, and Preliminary Results from the EARLY ALLIANCE Prevention Trial. *American Journal of Preventive Medicine* 2001;20(1S)

8. Sprange K, Mountain GA, Brazier J, et al. Lifestyle Matters for maintenance of health and wellbeing in people aged 65 years and over: study protocol for a randomised controlled trial. *Trials* 2013;14:302-02. doi: 10.1186/1745-6215-14-302

9. Clark F, Azen S, Zemke R, et al. Occupational Therapy for Independent-Living Older Adults. *Journal of the American Medical Association* 1997;278(16):1321-6.

10. Clark F, Jackson J, Carlson M, et al. Effectiveness of a lifestyle intervention in promoting the well-being of independently living older people: results of the Well Elderly 2 Randomised Controlled Trial. *Journal of Epidemiology & Community Health* 2011

11. Moncher F, Prinz R. Treatment fidelity in outcome studies. *Clinical Psychology Review* 1991;11:247-66.

12. Borrelli B. The assessment, monitoring, and enhancement of treatment fidelity in public health clinical trials. *Journal of Public Health Dentistry* 2011;71:S52-S63.

13. Datta J, Petticrew M. Challenges to evaluating complex interventions: a content analysis of published papers. *BMC Public Health* 2013;13:568. doi: 10.1186/1471-2458-13-568 [published Online First: 2013/06/14]

14. Bandura. A. Self-efficacy: The exercise of control.: New York: Freeman 1997.

15. Luszczynska A, Gutierrez-Dona B, Schwarzer R. General self-efficacy in various domains of human functioning: Evidence from five countries. *International Journal of Psychology* 2005;40(2):80-89.

16. Hasson H, Blomberg S, Duner A. Fidelity and moderating factors in complex interventions: a case study of a continuum of care program for frail elderly people in health and social care. *Implement Sci* 2012;7:23. doi: 10.1186/1748-5908-7-23 [published Online First: 2012/03/23]

17. Medical Research Council. Developing and evaluating complex interventions: new guidance. London: MRC; 2008

For peer review only

18. Carroll C, Patterson M, Wood S, et al. A conceptual framework for implementation fidelity. *Implement Sci* 2007;2:40. doi: 10.1186/1748-5908-2-40 [published Online First: 2007/12/07]

19. Hawe P, Shiell A, Riley T. Complex interventions: how “out of control” can a randomised controlled trial be? *BMJ* 2004;328(7455):1561-63. doi: 10.1136/bmj.328.7455.1561

20. Craig C, Mountain G. Lifestyle Matters: an occpational approach to healthy ageing: Bicester: Speechmark 2007.

21. National Institute for Health and Clinical Excellence (NICE). Public Guidance 6. Behaviour change: general approaches 2007 [Available from: http://guidance.nice.org.uk/PH6/Guidance/pdf/English.

22. Mountain G, Mozley C, Craig C, et al. Occupational therapy led health promotion for older people: feasibility of the lifestyle matters programme. *British Journal of Occupational Therapy* 2008;71(10):406-13.

23. Pope C, Mays N. Reaching the parts other methods cannot reach: an introduction to qualitative methods in health and health services research. *BMJ : British Medical Journal* 1995;311(6996):42-45.

24. McHugh ML. Interrater reliability: the kappa statistic. *Biochem Med (Zagreb)* 2012;22(3):276-82. [published Online First: 2012/10/25]

25. Gale N, Heath G, Cameron E, et al. Using the framework method for the analysis of qualitative data in multi-disciplinary health research. *BMC Med Res Methodol* 2013;13(117)

26. Ritchie J, Spencer L. Qualitative data analysis for applied policy research: The Qualitative Researcher's Companion: SAGE Publications 2002.

27. DG A. Practical statistics for medical research: London: Chapman and Hall 1991.

28. Duncan EAS, Nicol MM. Subtle Realism and Occupational Therapy: An Alternative Approach to Knowledge Generation and Evaluation. *The British Journal of Occupational Therapy* 2004;67(10):453-56. doi: 10.1177/030802260406701006

For peer review only - http://bmjopen.bmj.com/site/about/guidelines.xhtml

29. Masterson-Algar P, Burton CR, Rycroft-Malone J, et al. Towards a programme theory for fidelity in the evaluation of complex interventions. *J Eval Clin Pract* 2014;20(4):445-52. doi: 10.1111/jep.12174 [published Online First: 2014/05/21]

30. Resnick B, Inguito P, Orwig D, et al. Treatment Fidelity in Behaviour Change Research: a case example. *Nursing Research* 2005;54(2):139-43.

31. Moore G, Audrey S, Barker M, et al. Process evaluation in complex public health intervention studies: the need for guidance. *J Epidemiol Community Health* 2014;68(2):101-2. doi: 10.1136/jech-2013-202869 [published Online First: 2013/09/12]

32. Hoddinott P, Allan K, Avenell A, et al. Group interventions to improve health outcomes: a framework for their design and delivery. *BMC Public Health* 2010;10(800)

For peer review only

Table 1: Lifestyle Matters fidelity assessment framework and results

Element	Fidelity criteria	Fidelity standard	Standard met	Evidence
**Trial Design**				
Comparable treatment	All participants received the same programme tailored to the needs of the group/ setting.	• 16 weekly meetings offered to all participants. Attendance recorded.	Yes	• Trial documentation e.g. Information sheet.<br>• 176 (11x16) weekly registers recorded.
		• 4 individual sessions offered to all participants. Attendance recorded.	Yes	• Trial documentation e.g. Information sheet.<br>• 443 individual sessions offered, 143 taken-up.
Risk to implementation	Plan for potential issues that could affect the delivery of the lifestyle matters programme.	• A range of recruitment strategies will be implemented.	Yes	• Methods used included GP mail-outs, direct referrers and posters/leaflets.
		• A range of days and times for meetings offered, from which participants can choose.	Yes	• Facilitators worked flexibly.<br>• Groups took place on various days of the week and times of day (AM/PM) .
		• Undertake three recruitment cycles in three geographical areas, to prevent saturation.	Yes	• Recruitment conducted in multiple areas at each site.
**Monitoring provider training**				
Standardised training and facilitator skill acquisition	All facilitators receive the same training tailored to the group/setting and engage with the training in a similar way.	• Completion of training exercise by facilitators.	Yes	• Observation by researchers of participants completing role playing exercises and group work.
		• Training delivered by the same trainer.	Yes	• Same trainer delivered all training.
		• Manual/CD-Rom provided to all trainees.	Yes	• Manual (or copy) and CD provided to all trainees.
		• Observation of the training session by two researchers using a content checklist	Yes	• Observation by two researchers of skill transference using content checklist.<br>• Checklist coding developed to observe 'All' participants.
**Monitoring intervention delivery**				
Standardised delivery	All facilitators using the same techniques and content from the programme.	• Observation using a content checklist by two researchers	Yes	• Observation completed by two researchers.
		• 75% of opportunities for completing goal setting are recorded (for individual and group).	No	• Participants found concept of 'goal setting' difficult to understand. 'Goal setting' sheets provided in programme rarely used. Discussion of 'Planning' and 'Wishes' preferred.
		• Range of materials from the Lifestyle Matters programme received by all participants.	Yes	• Observation of manual materials used during recorded group meetings.<br>• Use of materials discussed by participants during qualitative interviews.
		• Facilitators maintain reflective diaries.	Yes	• Used as part of supervision sessions, confirmed by facilitators and supervisors.
		• Registers completed for weekly meetings and individual sessions.	Yes	• Registers completed and recorded on the trial database.
		• Participant and facilitator semi-structured interview topic guide.	Yes	• Interviews conducted with all facilitators at the end of cycle 1 and cycle 3. Interviews conducted with 10% sample of participants at the end of their programme attendance.
		• All participants receive certificate of attendance/achievement.	Yes	• Copies of certificates given to all participants across both sites.
		• Facilitators meet the NHS Band 4 equivalent job description	Yes	• Facilitators selected for interview based on meeting essential criteria of job description.
Minimise drift in skills/ delivery	Adherence to training content and delivery over the 3 cycles of the intervention	• Observation using a content checklist by two researchers	Yes	• Observation by two observers.
		• OT supervisor protocol.	Yes	• Protocol written and provided to supervisors and facilitators prior to the programme start.
		• Each facilitator will attend between 8 and 16 supervision sessions in total of which half should be delivered face-to face.	Yes	• Supervisor attendance records provided for two facilitators (consent obtained).<br>• Face-to-face contact recorded for one facilitator for 12 months. Three facilitator's supervision started as face-to-face moving to a combination with telephone contact.
**Monitoring receipt of intervention**				
Participant attendance and engagement	Recording participants weekly attendance<br><br>All participants taking part in the meetings and activities<br><br>Impact of intervention on participant in terms of wellbeing	• Registers completed by facilitator for weekly meetings and individual sessions where arranged.	Yes	• All registers completed and recorded.<br>• Interviews demonstrated that most participants took part in the programme, although not all joined every activity mainly due to health or mobility issues.
		• 75% of opportunities for completing goal setting are recorded (individual and group).	No	• Concept of 'goal setting' difficult to understand by participants. 'Planning' or 'Wishes' preferred.
		• Participant and facilitator semi-structured interview topic guide.	Yes	• All Facilitators interviewed at cycle 1 and cycle 3.<br>• 10% sample of participants interviewed.<br>• Participant behaviours observed during videoed sessions.
		• PROMS.	Awaiting results	• Completed PROMS recorded on trial database.

For peer review only

Table 2: Sample of video recorded group meetings

Site	Wave	Lifestyle Matters Group	Programme Week (1-16) videoed	No of Facilitators present	Participants attending/ registered
North England	1	1	6	2	10/13 (77%)
			10	2	11/13 (85%)
North Wales	1	1	4	2	7/10 (70%)
			10	2	7/9† (78%)
North England	3	6*	7	2	6/8 (75%)
			15	2	7/8 (88%)
North Wales	3	5*	4	2	8/9 (89%)
			10	2	7/9 (78%)

*North England site completed six Lifestyle Matters groups and North Wales completed five

†Participant withdrew

For peer review only

Table 3: Training fidelity scores by 'Trainer', 'Trainee' and total

	Agreed Score	Coder 1	Coder 2	Median
**Trainer score**	30/30 (100%)	30/30	30/30	30
**Trainee score**	43/57 (75.4%)	43/57	47/57	45
**Total**	73/87 (83.9%)	73/87	77/87	75
**Percentage of Total**		83.9%	88.5%	86.2%

For peer review only

Table 4: Fidelity scores for 'Facilitator performance'

	Facilitator score /out of 48							
**Wave 1**	**(Site A) Group 1**				**(Site B) Group 1**			
	**Rater 1**	**Rater 2**	**Agreed score**	**%**	**Rater 1**	**Rater 2**	**Agreed score**	**%**
Vid 1	38	33	37/48	**77.0%**	38	39	39/48	**81.2%**
Vid 2	22	28	25/48	**52.0%**	44	44	42/48	**87.5%**
**Wave 3**	**(Site A) Group 6**				**(Site B) Group 5**			
	**Rater 1**	**Rater 2**	**Agreed score**	**%**	**Rater 1**	**Rater 2**	**Agreed score**	**%**
Vid 1	44	44	44/48	**91.6%**	30	34	30/48	**62.5%**
Vid 2	46	45	44/48	**91.6%**	33	35	34/48	**70.8%**

For peer review only

Table 5: Fidelity scores for 'Group members'

	Group Member score /out of 39							
**Wave 1**	**(A) Group 1**				**(B) Group 1**			
	**Rater 1**	**Rater 2**	**Agreed score**	**%**	**Rater 1**	**Rater 2**	**Agreed score**	**%**
Vid 1	34	33	33/39	**84.6%**	37	35	38/39	**97.4%**
Vid 2	11	18	16/39	**41.0%**	30	29	29/39	**74.3%**
**Wave 3**	**(A) Group 6**				**(B) Group 5**			
	**Rater 1**	**Rater 2**	**Agreed score**	**%**	**Rater 1**	**Rater 2**	**Agreed score**	**%**
Vid 1	33	31	33/39	**84.6%**	23	28	24/39	**61.5%**
Vid 2	36	36	36/39	**92.3%**	26	26	26/39	**66.6%**

For peer review only

Additional file 1: Lifestyle Matters training delivery observation checklist

**TRAINER**	
**Domain**	**Criteria**
Trainer	1. The trainer is the same for all training
	2. The trainer is an author of the programme
	3. Demonstrates a range of group work skills (didactic teaching, peer sharing, interactive activities, active experimentation etc)
Materials	4. PowerPoint presentations and materials directly relate to the goals of the training
	5. Provides a CD of training content and materials to all trainees
Tailoring	6. Training is tailored to recipients' skill level, experience or work background
	7. Training is tailored in response to the observed needs of the trainees, and feedback from the trainees during the sessions
Content	8. The goals of the training are covered
	9. Examples of the core themes and examples of subsidiary themes are covered
	10. Mandatory themes are covered:<br>• A celebration of achievements<br>• Activity and health<br>• Endings
**TRAINEES**	(Performance in discussion, role play, problem solving, expressive exercises, reflective exercises shared, written tests)
**Domain**	**Criteria**
Understanding	11. Critically discuss demographic changes and the concept of active ageing
	12. Discuss the evidence for Lifestyle matters
	13. Identify the ideas that are key to Lifestyle Matters, including<br>• enabling group members to do things for themselves<br>• transferring skills from the safety of the group to their wider lives
	14. Discuss the content of the Lifestyle Matters manual and how to apply it
Self -awareness	15. Share their prior expertise, hopes and expectations of the training
	16. Share their own perceptions of ageing and how this may impact on the programme
	17. Reflect on and share their own facilitation style and skills
Skill acquisition	18. Use the LifeStyle Matters manual to practice planning sessions within themes
	19. Identify how to locate and access community resources
	20. Respond to potential scenarios, using creative problem solving and considering individual needs (including risk taking)
Group work	21. Understand and demonstrate a range of group work skills (didactic teaching, peer sharing, interactive activities, active experimentation etc)
	22. Understand and demonstrate how to manage group dynamics
	23. Understand and demonstrate an enabling style of group facilitation
	24. Demonstrate how to tailor the intervention to the needs and choices of group members
Individual sessions	25. Explore the value of 1:1 sessions and potential activities to use in these
Group outings	26. Explore the value or outings and how to help group members to plan them
Reflection & evaluation	27. Explore reflective practice and discuss its value
	28. Practice evaluating and planning actions
Supervision	29. Discuss the value and principles of supervision

For peer review only

Additional file 2: Lifestyle Matters intervention delivery video checklist

**FACILITATOR**	
**Domain**	**Criteria**
Resources	1. Two facilitators deliver
Personal qualities	2. Shows warmth and empathy
	3. Listens actively and respectfully
Enabling	4. builds on the assets, skills and abilities that each person brings
	5. Encourages the group to make choices
Group work skills	6. Facilitates communication and interaction (to ground rules?)
	7. Draws in quiet members of the group
	8. Facilitates peer exchange and guidance, and peer modelling
	9. Manages space, time and opportunity effectively
	10. Facilitates the members to explore differences between them, or conflict and resolution
	11. Builds mutual support and group cohesion
Goals and needs	12. Balances meeting individual needs and goals and group needs
	13. Balances sufficient challenge in activities with ensuring they are achievable
	14. Tailors activities to the needs of members
Content	15. Focuses on the themes in the programme, or new themes chosen by group members
	16. Uses group activities, not just discussion (activities from the manual or from elsewhere)
	17. With members, selects and plans group outings that explore and extend the themes in the programme
**GROUP MEMBERS**	
**Domain**	**Criteria**
Understanding	18. The relationship between activity, health and aging, or one of the other themes from the programme
Occupational self analysis	19. Analyse their own daily occupations (their significance, impact on health, components) and make choices
	20. Sharing and reflecting on experiences of participating in activities (Occupational storytelling)
Goal setting	21. Own goals identified within the group and within activities
Grading and layering	22. Grading and layering of activities to constantly revisit and extend skills and build confidence
Mutual support	23. Show mutual appreciation and support
Creativity	24. Practice any of: self expression, innovation, problem solving and adaptation within the safety of the group
	25. Engage in enjoyable and stimulating activities
Older people as experts	26. Acknowledge and appreciate their own skills and how they can contribute to the group
	27. Bring in and share their own skills and knowledge
	28. Group members in turn take leadership or contribute ideas
Transfer to everyday life	29. build skills, solutions or confidence within the group
	30. Report the transferring of skills, solutions or confidence from the group to wider life
Change over time	31. Plans for individuals are taken forward, whilst respecting each person's need to balance opportunities and risk
Confidence and hope	32. Achievements are celebrated
	33. Adventures and dreams are welcomed

For peer review only

BMJ Open

Evaluation of intervention fidelity in a randomised controlled trial of a complex psychosocial intervention: Lifestyle Matters

Journal:	*BMJ Open*
Manuscript ID	bmjopen-2020-043478.R1
Article Type:	Original research
Date Submitted by the Author:	27-Jan-2021
Complete List of Authors:	Sprange, Kirsty; University of Nottingham, Nottingham Clinical Trials Research Unit, University Park, NG7 2RD<br>Mountain, Gail; University of Bradford, Centre for Applied Dementia Studies, Faculty of Health Studies, Richmond Road, Bradford, BD7 1DP<br>Craig, Claire; Sheffield Hallam University, Lab4Living, Furnival Building, 153 Arundel Street, Sheffield, S1 2NU
**Primary Subject Heading**:	Research methods
Secondary Subject Heading:	Geriatric medicine
Keywords:	Dementia < NEUROLOGY, GERIATRIC MEDICINE, Clinical trials < THERAPEUTICS

SCHOLARONE™
Manuscripts

I, the Submitting Author has the right to grant and does grant on behalf of all authors of the Work (as defined in the below author licence), an exclusive licence and/or a non-exclusive licence for contributions from authors who are: i) UK Crown employees; ii) where BMJ has agreed a CC-BY licence shall apply, and/or iii) in accordance with the terms applicable for US Federal Government officers or employees acting as part of their official duties; on a worldwide, perpetual, irrevocable, royalty-free basis to BMJ Publishing Group Ltd ("BMJ") its licensees and where the relevant Journal is co-owned by BMJ to the co-owners of the Journal, to publish the Work in this journal and any other BMJ products and to exploit all rights, as set out in our licence.

The Submitting Author accepts and understands that any supply made under these terms is made by BMJ to the Submitting Author unless you are acting as an employee on behalf of your employer or a postgraduate student of an affiliated institution which is paying any applicable article publishing charge ("APC") for Open Access articles. Where the Submitting Author wishes to make the Work available on an Open Access basis (and intends to pay the relevant APC), the terms of reuse of such Open Access shall be governed by a Creative Commons licence – details of these licences and which Creative Commons licence will apply to this Work are set out in our licence referred to above.

Other than as permitted in any relevant BMJ Author's Self Archiving Policies, I confirm this Work has not been accepted for publication elsewhere, is not being considered for publication elsewhere and does not duplicate material already published. I confirm all authors consent to publication of this Work and authorise the granting of this licence.

For peer review only

Evaluation of intervention fidelity in a randomised controlled trial of a complex psychosocial intervention: Lifestyle Matters

Authors

Kirsty Sprange, University of Nottingham, Nottingham Clinical Trials Unit, University Park, NG7 2RD, UK. kirsty.sprange@nottingham.ac.uk (Corresponding author) ORCiD 0000-0001-6443-7242

Gail A Mountain University of Bradford, Centre for Applied Dementia Research, Faculty of Health Studies, Richmond Road, Bradford, BD7 1DP, UK g.mountain@bradford.ac.uk ORCiD 0000-0002-5417-7691

Claire Craig, Sheffield Hallam University, Lab4Living, Furnival Building, 153 Arundel Street, Sheffield, S1 2NU, c.craig@shu.ac.uk ORCiD 0000-0002-3475-3292

For peer review only

Abstract

Objectives: Robust research of complex interventions designed to promote mental wellbeing in later life is required to inform service development. An essential component is ensuring that such interventions are delivered as intended. This paper presents a detailed description of the design and implementation of a fidelity assessment within a trial of one such intervention (Lifestyle Matters). The findings help to explain the trial results and also inform the design of embedded fidelity assessments within future evaluations of complex interventions.

Design: We conducted a comprehensive mixed method fidelity assessment embedded as part of a multi-centre pragmatic, parallel group randomised controlled trial. A conceptual fidelity framework was developed from the Behavior Change Consortium framework, underpinned by mechanisms to facilitate behaviour change. From this the fidelity assessment was designed. The resulting instrument assessed the following parameters: intervention design, training, supervision; and delivery, receipt and enactment of the intervention.

Intervention: The Lifestyle Matters intervention uses an occupational approach to healthy ageing with the aim of improving and sustaining mental wellbeing. It incorporates a mix of facilitated group meetings and individual sessions to enable participants to redesign their lifestyle.

Results: The fidelity assessment demonstrated that the intervention was delivered as per protocol and was tailored to meet individual needs. There was substantial inter-rater agreement for training; and group member performance 0.72; and moderate agreement for facilitator performance 0.55. It was not possible to determine whether small declines seen in facilitator performance were due to facilitator drift or moderating factors such as group dynamics or participant characteristics.

Conclusions: The assessment methods adequately measured criteria identified as being significant indicators of fidelity. Adherence during training, delivery and supervision was good. The subjective nature of identification and rating observed behaviours was the main challenge. Future research should explore alternative methods of assessing fidelity in trials of complex interventions.

For peer review only - http://bmjopen.bmj.com/site/about/guidelines.xhtml

Trial registration: ISRCTN67209155

Keywords: Fidelity assessment, complex intervention, dementia

Strengths and Limitations of this study

- This study demonstrated that it is feasible to design a fidelity assessment framework and develop bespoke assessment tools to evaluate a complex multi-component psychosocial intervention.
- The extent of the assessment was limited by the methods used, for example it was not appropriate or even ethical to undertake observations of activities in the community.
- The validity of the findings is reliant on whether bespoke assessment tools are sensitive enough to identify the complex constructs within this type of intervention.

Background

An increase in long term conditions within the global population combined with increased longevity has led to interest in how tailored psychosocial interventions might enable individuals to retain physical and mental wellbeing in the extended lifespan [1 2]. UK guidance has identified the need for multi-component, tailored individual and group based preventative interventions which promote health and wellbeing through occupation and physical activity [3]. The benefits of social networking and group interventions involving activity targeting social isolation and loneliness has also been identified[4] but there is a limited range of recommended interventions. The challenges of associated research include intervention development and evaluation of intervention acceptability, followed by robust testing to determine intervention effectiveness [3]. Robust testing should include an assessment of intervention fidelity to assess the extent to which the intervention is delivered as intended.

Fidelity assessment and maintenance of fidelity [5 6] enables researchers to demonstrate with greater confidence that study results were due to the intervention and not to other confounding factors. Additionally observing causal mechanisms, how they produce change in a particular setting and how this context can affect implementation is essential for effective intervention implementation [7] and for promulgation of evidence based practice [8]. It is acknowledged that using multiple methods as part of a fidelity assessment can enhance validity and reliability of complex interventions [6]. Development and application of such

For peer review only - http://bmjopen.bmj.com/site/about/guidelines.xhtml

multi-method fidelity assessments in complex multi-component interventions have been demonstrated as feasible [9 10].

A pragmatic randomised controlled trial (RCT) with an embedded fidelity assessment was conducted to evaluate a complex manualised intervention, Lifestyle Matters, which was created to promote the mental wellbeing of community living older people [11]. Lifestyle Matters was developed for the UK setting from a US intervention, Lifestyle Redesign which was found to promote physical and mental health and wellbeing, occupational functioning and life satisfaction in older adults [12 13]. As reported in [11], the trial fully adhered to Consort guidelines.

Fidelity assessment for the Lifestyle Matters study included an evaluation of facilitator training and intervention delivery against pre-determined criteria; and a qualitative exploration of the mechanisms that mediated or moderated lifestyle change in study participants; thereby identifying causal mechanisms associated with implementation [11 14].

There is some debate about the benefits or otherwise of the manualisation of complex interventions. It has been observed that manaualisation can reduce variance in intervention delivery thereby enhancing fidelity [15]. Alternatively it can promote a generic approach which stifles flexibility, inhibits facilitator creativity and ignores the individual needs of participants [8 16]. As described below, Lifestyle Matters is underpinned by core principles but also promotes participant choice.

MRC guidance recommends that complex intervention design and associated fidelity assessment frameworks are developed from the underlying intervention theory[17 18]. Lifestyle Matters is underpinned by social cognitive theory. There is a focus upon promotion of self-management by participants [19], and in particular upon developing and sustaining self-efficacy as this is known to be a protective factor in improving motivation and emotional wellbeing [20]. Intervention theory is located in the promotion of self-efficacy [21] as well as a model that proposes that the transactional relationships between the person, environment and activities influence occupational performance [22]. The intervention guides participants to identify their own personal goals and work towards them. They are encouraged to use peer support through the group and support from facilitators in both group and individual sessions to actively overcome barriers to meaningful occupation within the community.

For peer review only - http://bmjopen.bmj.com/site/about/guidelines.xhtml

Interventions like Lifestyle Matters will have core components such as number of group and individual sessions and structured manual topics. However adapting a complex intervention to meet the needs of individual participants is important [23 24]. The Lifestyle Matters intervention provides a content and delivery structure, which is both flexible and readily tailored to the needs of participants. Therefore a range of methods and tools to assess and evaluate the fidelity of this tailored intervention were required. This was important as the application of measures to assess intervention adaptation can improve our understanding of how different components can impact upon delivery and receipt in context [25 26].

This paper describes the fidelity assessment conducted for the Lifestyle Matters study and presents the findings from analysis of facilitator training and supervision, intervention delivery and receipt. The findings are then discussed in the context of intervention implementation.

Trial design

The study was a pragmatic, two-arm, parallel group, individually randomised controlled trial designed to determine the population benefit of an occupation based intervention for people aged 65 years or older [27]. A total of 288 participants were randomised between 14 August 2012 and 19 April 2013 (18 couples and 252 individuals); 145 and 143 were allocated to the intervention and control groups, respectively. The intervention was delivered in one city in the north of England and in North Wales. Participants were eligible if they had reasonable cognitive function to participate in the intervention as evidenced by a score of 0 to 7 on the Six Item Cognitive Impairment Test (6CIT) [28]. They were also living independently or in sheltered accommodation, alone or with others and able to converse in English or Welsh. An associated fidelity assessment and qualitative sub-study were conducted to evaluate facilitator training and supervision; intervention delivery; and explore mechanisms that mediate or moderate lifestyle change by participants.

Patient and public involvement

Patients and members of the public were involved at all stages from the development of the intervention during feasibility testing through to trial design and conduct. We received input from older adults on the design of the trial materials and in the oversight of the delivery of the study through PPI representation on trial oversight groups. The main trial results were

For peer review only

disseminated to participants using materials suitable for a non-specialist audience and shared with the wider public, clinical and academic colleagues through events and publications.

The Lifestyle Matters intervention

The intervention involves volunteers taking part in sixteen facilitated sequential weekly group meetings with up to 15 others and in four individual sessions with one of the facilitators to pursue individual goals [29]. During group meetings participants are encouraged to think about and engage in discussion and activities to explore the relationship between occupation and health, selecting from a menu of potential topics. A crucial element is going into the community to try out new or neglected skills with support from each other and from the facilitators; at least four sessions should involve such activities.

Group meetings are approximately two hours in duration at a convenient community venue. The first group sessions are structured to cover key topics from the manualised intervention. This provides a foundation for facilitators to maintain adherence to the intervention and for both facilitators and participants to work together on future sessions using the same model. Short breaks of no more than two weeks can be included to accommodate statutory holidays.

Individual sessions are an important intervention component and are specifically directed by the individual's goals and preferences. The first session concentrates on the participant-facilitator relationship. The remaining three sessions are built around the participant and what they want to achieve, using the intervention to assist them to execute this.

Facilitators and facilitation

Facilitators with the necessary skill set and qualities were recruited to deliver the intervention as intended [16]. They had to have prior experience of working with groups and with older people. A requirement of intervention delivery is for two facilitators to be present during each group and to share working with participants during individual sessions. Facilitators could be involved in the delivery of up to three intervention groups of 8-16 participants at any one time (including associated individual sessions).

Facilitator Supervision

Supervision was provided by appropriately experienced Occupational Therapists who also attended facilitator training alongside the facilitators. A protocol was created to enable

For peer review only - http://bmjopen.bmj.com/site/about/guidelines.xhtml

provision of consistent and appropriate supervision across and within sites. Regular one-to-one supervision was recommended on a weekly basis at a mutually convenient time and place. Preferably face-to-face but with distance supervision being an option if appropriate. Joint supervision was also deemed acceptable if the individual supervisory needs of facilitators had been met.

Training

A two day intensive training course was delivered jointly to all facilitators and supervisors before intervention delivery commenced, thereby ensuring that everyone received the same information and training experience to equip them to deliver the intervention and improve fidelity [30].

Training content included presenting the rationale and theory behind the intervention and intervention components [16]. The training then mirrored delivery of Lifestyle Matters intervention and manual [31] in that it involved;

- understanding the importance of taking a personalised and tailored approach towards participants at all times [32].
- didactic sessions to explain the programme and its ethos.
- peer sharing; providing the opportunity to share narratives, explore experience and attitudes and reflect on the relationship that can develop between participants and between facilitators and participants and between supervisor and facilitators.
- undertaking active experimentation, thereby testing out ideas and concepts.
- exploring communication skills through role playing, vignettes and group discussions.

The two-day training was video recorded to give facilitators an idea of how they and their participants might experience this for fidelity assessment and smooth out any challenges with using the recording equipment.

All facilitators and supervisors received a copy of the manual and an accompanying CD-Rom with downloadable resources for intervention delivery [31].

For peer review only

Materials and methods

Fidelity assessment framework

A comprehensive fidelity assessment framework specific to the intervention was developed (see Table 1) based on the Behaviour Change Consortium (BCC) recommendations and NICE guidance on behaviour change [5 33]. Fidelity assessment and quality assurance parameters centred on the BCC five recommended criteria: intervention design, training, delivery, receipt and enactment. For each criterion, programme objectives based on the Lifestyle Matters manualised intervention and training were discussed and agreed by two senior researchers. A description for each standard was then developed and methods to demonstrate fidelity to each standard identified (see Table 1).

Table 1: Lifestyle Matters fidelity assessment framework and results

Element	Fidelity criteria	Fidelity standard	Standard met	Evidence
**Trial Design**				
Comparable treatment	All participants received the same programme tailored to the needs of the group/ setting.	• 16 weekly meetings offered to all participants. Attendance recorded.	Yes	• Trial documentation e.g. Information sheet.<br>• 176 (11x16) weekly registers recorded.
		• 4 individual sessions offered to all participants. Attendance recorded.	Yes	• Trial documentation e.g. Information sheet.<br>• 443 individual sessions offered, 143 taken-up.
Risk to implementation	Plan for potential issues that could affect the delivery of the lifestyle matters programme.	• A range of recruitment strategies will be implemented.	Yes	• Methods used included GP mail-outs, direct referrers and posters/leaflets.
		• A range of days and times for meetings offered, from which participants can choose.	Yes	• Facilitators worked flexibly.<br>• Groups took place on various days of the week and times of day (AM/PM) .
		• Undertake three recruitment cycles in three geographical areas, to prevent saturation.	Yes	• Recruitment conducted in multiple areas at each site.
**Monitoring provider training**				
Standardised training and facilitator skill acquisition	All facilitators receive the same training tailored to the group/setting and engage with the training in a similar way.	• Completion of training exercise by facilitators.	Yes	• Observation by researchers of participants completing role playing exercises and group work.
		• Training delivered by the same trainer.	Yes	• Same trainer delivered all training.
		• Manual/CD-Rom provided to all trainees.	Yes	• Manual (or copy) and CD provided to all trainees.
		• Observation of the training session by two researchers using a content checklist	Yes	• Observation by two researchers of skill transference using content checklist.<br>• Checklist coding developed to observe 'All' participants.
**Monitoring intervention delivery**				
Standardised delivery	All facilitators using the same techniques and content from the programme.	• Observation using a content checklist by two researchers	Yes	• Observation completed by two researchers.
		• 75% of opportunities for completing goal setting are recorded (for individual and group).	No	• Participants found concept of 'goal setting' difficult to understand. 'Goal setting' sheets provided in programme rarely used. Discussion of 'Planning' and 'Wishes' preferred.
		• Range of materials from the Lifestyle Matters programme received by all participants.	Yes	• Observation of manual materials used during recorded group meetings.<br>• Use of materials discussed by participants during qualitative interviews.
		• Facilitators maintain reflective diaries.	Yes	• Used as part of supervision sessions, confirmed by facilitators and supervisors.
		• Registers completed for weekly meetings and individual sessions.	Yes	• Registers completed and recorded on the trial database.

For peer review only

		• Participant and facilitator semi-structured interview topic guide.	Yes	• Interviews conducted with all facilitators at the end of cycle 1 and cycle 3. Interviews conducted with 10% sample of participants at the end of their programme attendance.
		• All participants receive certificate of attendance/achievement.	Yes	• Copies of certificates given to all participants across both sites.
		• Facilitators meet the NHS Band 4 equivalent job description	Yes	• Facilitators selected for interview based on meeting essential criteria of job description.
Minimise drift in skills/ delivery	Adherence to training content and delivery over the 3 cycles of the intervention	• Observation using a content checklist by two researchers	Yes	• Observation by two observers.
		• OT supervisor protocol.	Yes	• Protocol written and provided to supervisors and facilitators prior to the programme start.
		• Each facilitator will attend between 8 and 16 supervision sessions in total of which half should be delivered face-to face.	Yes	• Supervisor attendance records provided for two facilitators (consent obtained).<br>• Face-to-face contact recorded for one facilitator for 12 months. Three facilitator's supervision started as face-to-face moving to a combination with telephone contact.
**Monitoring receipt of intervention**				
Participant attendance and engagement	Recording participants weekly attendance<br><br>All participants taking part in the meetings and activities<br><br>Impact of intervention on participant in terms of wellbeing	• Registers completed by facilitator for weekly meetings and individual sessions where arranged.	Yes	• All registers completed and recorded.<br>• Interviews demonstrated that most participants took part in the programme, although not all joined every activity mainly due to health or mobility issues.
		• 75% of opportunities for completing goal setting are recorded (individual and group).	No	• Concept of 'goal setting' difficult to understand by participants. 'Planning' or 'Wishes' preferred.
		• Participant and facilitator semi-structured interview topic guide.	Yes	• All Facilitators interviewed at cycle 1 and cycle 3.<br>• 10% sample of participants interviewed.<br>• Participant behaviours observed during videoed sessions.
		• PROMS.	Awaiting results	• Completed PROMS recorded on trial database.

Adapted from Bellg et al, 2004 [5]

Assessment tools

The underlying ethos of Lifestyle Matters and the tailored approach to delivery make fidelity assessment complex as many of the identified criteria are subjective. A range of quantitative and qualitative tools were therefore used in line with MRC recommendations for complex interventions [24], including attendance registers, observation checklists and interview schedules which were all piloted before application.

Attendance registers

Registers were used to record both group and individual session attendance. Using pilot study findings it was estimated that participants would need to attend at least half (eight) group meetings, to benefit from the intervention [34]. The number of individual sessions required to derive benefit was not set.

For peer review only - http://bmjopen.bmj.com/site/about/guidelines.xhtml

Development of observation checklists

Observation checklists were developed by two senior researchers who identified domains comprising the core skills and key criteria identified in the Lifestyle Matters manual, worksheets and training materials (see additional file 1). Criteria described the components and behaviours expected to be present during intervention delivery. Domains were divided into those associated with 'Trainer or Trainee performance' and those associated with 'Facilitator or Group member performance'.

A scoring system was devised. Several criteria were rated on a binary 'Yes' or 'No' scale for presence or absence. For the purpose of analysis if a criterion was present this was converted to a score of 3. If the criterion was not present this was converted to a score of 0.

Fidelity scores were calculated based on agreement of a final score between the two coders and the total was given a percentage score as follows. The scoring system was adapted from the principle set out in Borelli 2011, that 80-100% constitutes high fidelity [16]:

- 0- 60% Unsatisfactory
- 61-70% Satisfactory
- 71-80% Good
- 81-90% Very good
- 91-100% Excellent

To ensure consistency the same two researchers completed fidelity assessment activities and coding. The patient and public contributor for the project also contributed to the fidelity assessment analysis including reviewing and piloting observation checklists (as described below), as well as participant observation during training.

Assessment of the fidelity of training delivery

Participant observation, in which the researcher not only observes, but takes an active role in the setting [35], was selected to assess the fidelity of training delivery. The researchers therefore attended the training as delegates. During the training, sessions were independently rated by two researchers. There were also discussions between the trainer and researchers at the beginning and end of each day to review content and feedback from attendees. All scores

For peer review only - http://bmjopen.bmj.com/site/about/guidelines.xhtml

were then shared and reviewed for reliability of interpretation and coding against checklist criteria.

Assessment of the fidelity of intervention delivery

A purposive sample of eight video-recordings were taken of four of the 11 Lifestyle Matters groups to monitor implementation and adherence to the intervention and identify any facilitator drift over time. To reduce bias the same number of groups and group meetings from both sites were selected for assessment. Written consent to conduct video recordings was obtained from each participant and facilitators recorded the group meetings using simple compact video recorders.

One video of each of the four selected groups was recorded at approximately week four and again at around week 10 of intervention delivery although some flexibility in session choice was necessary to avoid out of venue activities (see Table 2). Observing two meetings per group; one at the beginning and one near the end enabled us to examine any learning effects and identify any facilitator drift [36].

Table 2: Sample of video recorded group meetings

Site	Wave	Lifestyle Matters Group	Programme Week (1-16) videoed	No of Facilitators present	Participants attending/ registered
North England	1	1	6	2	10/13 (77%)
			10	2	11/13 (85%)
North Wales	1	1	4	2	7/10 (70%)
			10	2	7/9† (78%)
North England	3	6*	7	2	6/8 (75%)
			15	2	7/8 (88%)
North Wales	3	5*	4	2	8/9 (89%)
			10	2	7/9 (78%)

*North England site completed six Lifestyle Matters groups and North Wales completed five

†Participant withdrew

Video content was then rated using the predetermined checklist and findings analysed to determine delivery and receipt of the intervention and any change over time.

During piloting of the intervention fidelity checklist, it became apparent that several of the assessment criteria could not be observed during the weekly group meetings. For example,

For peer review only - http://bmjopen.bmj.com/site/about/guidelines.xhtml

receipt and enactment of the intervention would occur in the participant's everyday life away from the group. It was deemed that either the criterion could not be present during every weekly meeting or it would be necessary to observe several meetings consecutively which was not planned. Further observations using the checklist to test the assumption found this to be consistent. The criteria deemed to be immeasurable were therefore excluded from the final analysis.

Due to practicalities, only the groups held within the meeting venue were observed. Given the importance of enactment of activities in the community this was a limitation.

We did not undertake observations of the individual sessions as these were deemed a safe space for participants to work with their facilitator on individual goals. These sessions could also involve activities in the community where it would be impractical and unethical for researchers to observe sessions.

Inter-rater reliability

Inter-rater reliability demonstrates whether available data accurately represents the variables being measured and the extent to which coders attribute the same score to the same variable [37]. Inter-rater reliability was calculated by two researchers independently coding video recordings and by scores being compared using the Kappa statistic, with a value of 0.61 and above indicating good inter-rater agreement.

- < 0.20 Poor
- 0.21-0.40 Fair
- 0.41-0.60 Moderate
- 0.61-0.80 Good
- 0.81-1.00 Very good

Semi-structured interviews

Semi-structured interviews were undertaken with all facilitators to explore views on what enables effective intervention facilitation, and the skills and competencies required to deliver the intervention and enable participants to act on it [29]. Interviews took place at the end of the first and third (final) wave of intervention delivery to identify any changes in experiences of delivery over time.

For peer review only - http://bmjopen.bmj.com/site/about/guidelines.xhtml

An interview topic guide was developed informed by the content of the training materials and intervention manual as well as knowledge gained from prior feasibility work [34].

A purposeful sample of 10% of participants were interviewed; selected from both sites and from each of the three waves of intervention delivery to elicit the range and nature of issues that influenced their experiences of the intervention. The sample was considered adequate to reach data saturation within resource limitations.

All transcripts were read for familiarisation and annotated for themes by the same two researchers (Fidelity lead and second researcher) using Framework analysis in NVivo [38 39]. For the purposes of the fidelity assessment, data from the interviews were used to identify cross cutting themes and explanatory factors for fidelity outcomes.

Results

The 'Fidelity Assessment Framework' including evidence for fidelity is presented in table 1.

Facilitators

All four facilitators remained the same throughout the trial at both sites. They were all female, younger than the youngest participant (65 years) and from a health or social care professional background.

Attendance

Eleven Lifestyle Matters interventions were delivered, six in one city in Northern England and five in North Wales. Four groups were selected for the fidelity assessment as previously described. Overall attendance at each was 70% or above (see table 2). The average number of out of venue activities conducted as a part of the group aspect of the intervention across all groups was four.

Training delivery

The two-day training was attended by all four facilitators (two from each site) and the two supervisors (one from each site).

For peer review only - http://bmjopen.bmj.com/site/about/guidelines.xhtml

Excellent fidelity was demonstrated for trainer performance with a score of 100% across all four domains.

Good fidelity was identified through a criterion score of 75.4% across eight domains for trainees. Overall training fidelity was very good with a total agreed criterion score for 'trainer performance' and 'trainee performance' of 83.9%.

Intervention delivery

Facilitator performance

Excellent or very good fidelity was achieved in four out of the eight recorded groups (see Table 3). Only one observed group (Wave 1, Site A, Video 2) demonstrated unsatisfactory fidelity (52%). The other three groups all showed either an increase or maintenance in fidelity scores.

Table 3: Fidelity scores for 'Facilitator performance'

	**Facilitator score /out of 48**							
**Wave 1**	**(Site A) Group 1**				**(Site B) Group 1**			
	**Rater 1**	**Rater 2**	**Agreed score**	**%**	**Rater 1**	**Rater 2**	**Agreed score**	**%**
Video 1	38	33	37/48	**77.0%**	38	39	39/48	**81.2%**
Video 2	22	28	25/48	**52.0%**	44	44	42/48	**87.5%**
**Wave 3**	**(Site A) Group 6**				**(Site B) Group 5**			
	**Rater 1**	**Rater 2**	**Agreed score**	**%**	**Rater 1**	**Rater 2**	**Agreed score**	**%**
Video 1	44	44	44/48	**91.6%**	30	34	30/48	**62.5%**
Video 2	46	45	44/48	**91.6%**	33	35	34/48	**70.8%**

Excellent or very good fidelity was demonstrated in three of the six assessment domains. Only one domain; 'Goals and Needs' was rated as being unsatisfactory. This required evidence of programme tailoring and how facilitators helped participants to select achievable activities with appropriate levels of challenge.

The decline in fidelity seen at Site A during Wave 1 (77% to 52%), could be explained by the facilitators arranging for two external speakers to deliver sessions on relaxation and physical health. During the meeting the facilitators largely left facilitation to the external speakers who used a didactic style, which did not invite discussion or tailoring to the needs of group members. It was deemed by both coders that the facilitators could have coached external speakers on how to deliver a session in accordance with the Lifestyle Matters ethos and could have interjected during the sessions. An outcome from this session was to provide facilitators

For peer review only - http://bmjopen.bmj.com/site/about/guidelines.xhtml

with further guidance on when and how to engage external speakers. This was delivered as an email to all facilitators ensuring they received consistent information.

A difference was also noted between the first and last waves at Site B where the assessed fidelity dropped from 87.5% to 70.8% but was still within the boundary set for satisfactory fidelity. Based on findings from the qualitative interviews, reasons for the reduction in scores could be explained by difficulties in time management to undertake planning and delivery of multiple consecutive groups as well as the challenges presented by the dynamics within groups.

Group member performance

Excellent or very good fidelity was demonstrated for four out of the eight recorded groups (see Table 4). Only one group (Wave 1, Site A) was rated as being unsatisfactory (41%) with this being related to the same issue described earlier for 'Facilitator performance'.

Table 4: Fidelity scores for 'Group members'

	**Group Member score /out of 39**							
**Wave 1**	**(A) Group 1**				**(B) Group 1**			
	**Rater 1**	**Rater 2**	**Agreed score**	**%**	**Rater 1**	**Rater 2**	**Agreed score**	**%**
Video 1	34	33	33/39	**84.6%**	37	35	38/39	**97.4%**
Video 2	11	18	16/39	**41.0%**	30	29	29/39	**74.3%**
**Wave 3**	**(A) Group 6**				**(B) Group 5**			
	**Rater 1**	**Rater 2**	**Agreed score**	**%**	**Rater 1**	**Rater 2**	**Agreed score**	**%**
Video 1	33	31	33/39	**84.6%**	23	28	24/39	**61.5%**
Video 2	36	36	36/39	**92.3%**	26	26	26/39	**66.6%**

Excellent or very good fidelity was demonstrated for three out of eight identified domains with unsatisfactory evidence for only one; 'Confidence and hope'. However, it became evident that ability to score some criteria was affected by the nature of the activity's participants engaged in.

Inter-rater reliability

Kappa scores were calculated using linear weights [40].

For the assessment of training fidelity, the inter-rater reliability between coders was Kappa = 0.72 (p <.0.001), 95% CI (0.478, 0.964).

Inter-rater reliability for overall fidelity to intervention delivery across sites was Kappa = 0.63 (p <0.001), 95% CI (0.543, 0.719).

Inter-rater reliability for facilitator domains across both sites was Kappa = 0.55 (p <0.001), 95% CI (0.432, 0.678). For group members inter-rater reliability was Kappa = 0.72 (p <0.001), 95% CI (0.598, 0.844).

Qualitative findings

Post group delivery Facilitator interviews

Analysis of facilitator interviews revealed that they all considered that they had become more confident, skilled, and flexible over time. Also, that their commitment to the ethos of the programme was maintained:

> "*We've actually grown in confidence…it feels a very different place to where we were at the beginning... far more skilled... allowing for that flexibility and spontaneity" (Facilitator D).*

Further training opportunities such as booster sessions or shadowing experienced facilitators were suggested to help consolidate knowledge of the intervention and improve fidelity further.

> *"…it would have been useful just to have…a follow up session just to see what kinds of things were coming up for people and have that, that kind of discussion" (Facilitator B).*

Regular self-review, using a group observation checklist was proposed by facilitators as a tool to enable them to reflect on delivery style and decision-making processes. (Facilitators were aware of the checklist as part of the fidelity work but did not have sight of the checklist).

> *"it may be more useful in future, if the facilitators could peer review themselves every three weeks against the checklist…scoring themselves and detailing an action against those that need to be achieved" (Facilitator A).*

One facilitator suggested that additional content in the training on how to avoid dependency relationships when facilitating using concrete examples case studies and written guidance would have been helpful.

For peer review only - http://bmjopen.bmj.com/site/about/guidelines.xhtml

"...what might breed relationships of dependency, what are the little things we [facilitators] don't realise we're doing, I think that might be something that could be added to the training" (Facilitator A)

The same facilitator thought that fostering and enabling independence was also important for the intervention and more time dedicated in the training was needed.

"...to process and assimilate all that..." (Facilitator A).

Another facilitator suggested that training should incorporate more on managing group dynamics and handling difficult people, and how to adapt sessions spontaneously when needed.

"...few trouble-shooting scenarios..." (Facilitator D).

One facilitator described modifying their approach to goal setting within the individual sessions where they felt participants had found the concept too formal or difficult to understand. Rather than talking about conscious goals they would integrate it subtly into the intervention.

"Interestingly you'd be approaching that same subject in a very subtle way over the weeks and weaving it into natural conversation, that was received a lot better..." (Facilitator 1).

Two facilitators reported levels of performance anxiety due to the session being recorded, which they felt may have hindered their delivery of the intervention.

"I think it is fair to say that being recorded has significantly affected the performance of the facilitators due to nerves of being recorded. As such I don't think the recordings reflect what has actually taken place within the groups over time. We tick many of the boxes on the checklist, but the recordings I feel won't pick a lot of this up" (Facilitator D).

Discussion

The framework

The fidelity assessment framework designed for this study provided a satisfactory structure for monitoring and evaluating adherence to the intervention over time. We were able to

For peer review only - http://bmjopen.bmj.com/site/about/guidelines.xhtml

demonstrate good fidelity to training as well as delivery of the group component of the intervention but were unable to observe all intervention components including the individual sessions and the out of venue activities. There were however several shortcomings to the framework approach[5] for this type of intervention. Assessment frameworks are designed to rate set criteria, which by definition should be the same for all observations. This is not necessarily true of complex interventions such as Lifestyle Matters that have tailoring as an underlying mechanism. To accommodate this, the underpinning ethos of the intervention along with the manual and training content provided the basis of the observation checklists[41]. This allowed for a range of observed behaviours to be considered in relation to set criteria instead of only one behaviour signifying presence.

Fidelity assessment methodology and adaptation

We recognised the adaptive nature of the intervention and the resultant complexity for fidelity assessment at the time of delivering this study. Current debate is indeed questioning whether fidelity and adaptation can co-exist[42]. Being able to determine the difference between intervention ineffectiveness or alternatively failure to deliver as intended can significantly impact the implementation outcome[43]. This intervention includes a number of interlinked adaptations; namely selection of different components from a menu of possibilities, the context, the characteristics and needs of the participant and the group they are a member of and the characteristics and abilities of facilitators. Whether it is possible to measure a range of adaptions for such tailored interventions, and how this would be achieved requires further consideration.

Coding

Coding performance can raise concerns. Expectations of both the facilitators and those rating performances must be considered and moderated. All those involved should try to avoid pre-occupation with fidelity assessment at the cost of intervention delivery[8]. Coders were not independent of the study, having attended the training as participant observers. Having the same knowledge and learning experience as those expected to deliver the intervention could enhance coder observations through greater understanding of observed behaviours and processes. Alternatively, using independent coders would reduce bias of unknown factors arising from personal knowledge of facilitators and increase the rigour of fidelity assessment. Overall we deduce that when the intervention is complex, ability to interpret what is

For peer review only

For peer review only - http://bmjopen.bmj.com/site/about/guidelines.xhtml

occurring is required if accurate observations are to be recorded[44]. Using researchers with knowledge or experience of the intervention as coders may therefore be preferable.

Training and competence

One of the main challenges to fidelity was the delivery of high quality in-depth training for a complex psycho-social intervention in just two days to a range of staff. Chances of variation in intervention delivery were deemed to lie in facilitator background and experience which are related to fidelity [6]. Evidence from the qualitative interviews also indicated that coverage of several core principles of the intervention including managing individual and group dynamics could have been improved during training. In this trial it was not possible for facilitators to give feedback on the training they had received but in real world implementation we would recommend including this.

The Lifestyle Matters manual and training are founded on 'occupational therapy' (OT) theory and practice and the facilitators were supervised by trained OTs. However, in pursuance of evidence that the intervention might be delivered by a range of staff, the recruited facilitators came from a variety of professional backgrounds including social work and mental health advocacy as well occupational therapy. Further customisation of training may be required to meet the needs of a wider range of health and social care staff.

Some deterioration of skills, or drift, is common post training [16]. Although no additional training or booster sessions were provided during this study, facilitators received regular supervision and occasionally received emailed 'study updates'. These communications (approved by the Trial Management Group) clarified details on intervention delivery to help reduce potential facilitator drift. Any emergent ethical concerns would have been immediately acted on if identified during the fidelity assessment. Although fidelity was generally found to be good, further training opportunities such as booster sessions or shadowing experienced facilitators might be incorporated in future studies to test whether additional training consolidates knowledge of the intervention and further improves fidelity. In addition the group observation checklist used to assess adherence and competence[15 45] to deliver the intervention could be used by facilitators as a tool to self-regulate and thereby enhance fidelity[46].

For peer review only - http://bmjopen.bmj.com/site/about/guidelines.xhtml

Facilitators and supervisors

It has been suggested that due to the key role of facilitators they should be cognisant of the impact of fidelity [6]. The importance and nature of the planned fidelity assessment was explained to the facilitators during the two-day preparatory training before they took part in the assessment. This was beneficial in providing reassurance about the process including the observations.

Even though facilitators were from a range of backgrounds and had different experiences of working with older people in the community, this did not appear to negatively impact on intervention delivery [36]. The facilitator job description and person specification provided a priori level of credentials required to provide some consistency across facilitators. However, the previous point about responsiveness to needs for training and support by those running the study should be maintained. This also applies to supervisors who are not experienced in delivering the programme.

A ‘Good fit’ between providers and participants can be key to successful intervention delivery [16]. All facilitators in this trial were female and younger than the youngest participant but this did not appear to impact on delivery. The supervisors were generally older and more experienced in delivering occupational therapy-based interventions, which could be drawn upon during supervision sessions.

Receipt of intervention

Fidelity to the intervention increased between waves for Site A, but gradually decreased for Site B. This could have been due to the dynamics of the groups that were recorded; challenges with the methods used to assess fidelity or may have been indicative of facilitator drift over time. The only factor that could be identified to explain facilitator drift at site B was the time pressure they expressed in trying to prepare and deliver the group sessions as well as trying to organise and conduct individual sessions. This explanation is commensurate with the slight decline seen in ‘Facilitator performance’ and suggests that additional training may have been beneficial.

Fidelity was also influenced by receipt of the intervention. For example, we expected to observe group and individual goal setting opportunities in intervention sessions. However, from the qualitative interviews it was evident that the majority of participants struggled to

For peer review only

engage with the concept of 'goal setting' and facilitators were required to make significant changes to their own understanding and how they presented the concept to participants[14]. Low fidelity therefore may not necessarily be associated with poor fidelity to delivery but may highlight a problem with the original intervention requirements, the nature of associated training and supervision, and subsequent criteria identified to evidence fidelity. Detailed analysis of feasibility study findings is therefore essential to establish clear baselines for behavioural interventions. Good design and piloting of tools is also important.

Practicalities and ethical considerations

The assessment only involved the video recording of a small number of weekly meetings out of the total provided and therefore it is inevitable that some facilitator and participant behaviours that may have affected intervention delivery were not observed. The assessment process also did not include observations of facilitators arranging and conducting individual sessions or out of venue activities which would have enhanced our understanding of the underlying intervention mechanisms. As previously described there were ethical considerations around participant confidentiality when assessing fidelity in a community setting. Consideration should therefore be given to how fidelity assessment can be undertaken within any given setting without interfering with the intervention [47]. How many, when and what type of sessions to record should be dependent on the complexity of the trial and availability of resources. For example, it may be prudent to monitor consecutive sessions to capture more information regarding participant receipt and enactment. Behaviour change may be more evident if an activity undertaken during one session can then be followed through to the next.

Psychosocial interventions are not designed to be passive; participants and facilitators bring their life experience, beliefs and expectations [36 48]. When determining intervention fidelity it is therefore important to consider how group dynamics and also environmental factors [49] impact not only on group composition but also group behaviour, especially when external factors to the intervention may be outside the control of those facilitating [48].

Conclusions

The outcomes from this fidelity assessment add to the growing literature on the use of fidelity assessment in trials and other studies of complex interventions. Psychosocial interventions that are flexible and offer a tailored approach in delivery may make fidelity assessment

For peer review only - http://bmjopen.bmj.com/site/about/guidelines.xhtml

difficult, but not impossible. Paradoxically the importance of undertaking thorough fidelity assessment is enhanced for complex interventions, which have a number of components. Despite the challenges, this study has demonstrated that by testing methods identified in a replicable framework, fidelity assessment can be carried out successfully.

Further research should explore the potential of different observational methods for monitoring fidelity in psycho-social community based complex trials and the impact of these methods on intervention delivery.

Abbreviations

Behaviour Change Consortium (BCC)

Randomized controlled trial (RCT)

Declarations

Ethics approval and consent to participate

The study was approved by the South Yorkshire Research Ethics Committee 12/YH/0101, the National Institute for Social Care and Health Research (NISCHR) Permissions Co-ordinating Unit in Wales, Betsi Cadwaladr University Health Board. Written informed consent was obtained from all participants.

Consent for publication

We obtained written informed consent for the participants who took part in the interviews via the Trial Consent Form under the following statement:

"I understand and give permission for group meetings or interviews that I may be asked to take part to be video/audio recorded and for the resulting data to be used in reports and publications including direct quotation."

This information is held as part of the archived record of the trial. Only anonymised non-identifiable data are used in this report as per written consent.

Availability of data and material

The datasets generated and analysed for this study will be available upon request from the authors.

For peer review only

Competing interests

GM and CC are the original authors of the published Lifestyle Matters manualised programme. No other authors have any competing interests.

Funding

The trial was funded through the Medical Research Council (MRC) Lifelong Health and Wellbeing (LLHW) programme, grant number G1001406, ISRCTN is 67209155. The views and opinions expressed in this paper are those of the authors and do not necessarily reflect those of the MRC. Primary Care Research Network (PCRN) funding was accessed to support recruitment activity in GP surgeries in Sheffield and NISCHR provided support in North Wales. The funder has reviewed and approved the trial design including methods of data collection, analysis and interpretation of data.

Author contributions

GM and CC obtained the funding. GM, KS and CC developed the research design, fidelity assessment framework and data collection tools. KS managed the research and undertook data collection. KS and GM analysed and interpreted the data. KS drafted the manuscript. All authors critically reviewed and approved the manuscript.

Acknowledgements

PPI contributors. Prof John Brazier, Dr Daniel Hind, Prof Stephen Walters, Dr Gill Windle, Prof Robert Woods (TMG) advised on the trial protocol including the process evaluation and fidelity assessment. Dr Pip Logan, Dr Jennifer Wenborn, Dr Linda Sheppard, Dr Fiona Goudie of the Trial Steering Committee (TSC) and Dr Mona Kanaan, Prof Avril Drummond and Dr Claire Ballinger of the Data Monitoring and Ethics Committee (DMEC) advised on and critically reviewed the trial protocol including the process evaluation and fidelity assessment.

References

1. McDermott O, Charlesworth G, Hogervorst E, et al. Psychosocial interventions for people with dementia: a synthesis of systematic reviews. *Aging & mental health*

For peer review only - http://bmjopen.bmj.com/site/about/guidelines.xhtml

2019;23(4):393-403. doi: 10.1080/13607863.2017.1423031 [published Online First: 2018/01/18]

2. Moniz-Cook E, Vernooij-Dassen M, Woods B, et al. Psychosocial interventions in dementia care research: the INTERDEM manifesto. *Aging & mental health* 2011;15(3):283-90. doi: 10.1080/13607863.2010.543665 [published Online First: 2011/04/15]

3. National Institute for Health and Care Excellence. Public health guideline [PH16]. Mental wellbeing in over 65s: occupational therapy and physical activity interventions 2008 [Available from: https://www.nice.org.uk/guidance/ph16 accessed 03Januray2018.

4. Government Office for Science. Foresight Mental Capital and Wellbeing: Making the most of ourselves in the 21st century 2008 [Available from: http://www.bis.gov.uk/assets/biscore/corporate/migratedD/ec_group/116-08-FO_b accessed January 2013.

5. Bellg A, Resnick B, Sharp Minicucci D, et al. Enhancing treatment fidelity in health behaviour change studies: Best practices and recommendations from the NIH Behaviour Change Consortium. *Health Psychology* 2004;23(5):443-51.

6. Spillane V, Byrne M, Byrne M, et al. Monitoring treatment fidelity in a randomised controlled trial of a complex intervention. *J Adv Nurs* 2007;60(3):343-52.

7. Craig P, Dieppe P, Macintyre S, et al. Developing and evaluating complex interventions: the new Medical Research Council guidance. *Bmj* 2008;337:a1655. doi: 10.1136/bmj.a1655 [published Online First: 2008/10/01]

8. Dumas J, Lynch A, Laughlin J, et al. Promoting Intervention Fidelity. Conceptual Issues, Methods, and Preliminary Results from the EARLY ALLIANCE Prevention Trial. *American Journal of Preventive Medicine* 2001;20(1S)

9. O'Malley KA, Qualls SH. Application of treatment fidelity in tailored caregiver interventions. *Aging & mental health* 2020;24(12):2094-102. doi: 10.1080/13607863.2019.1647134

10. Walton H, Tombor I, Burgess J, et al. Measuring fidelity of delivery of the Community Occupational Therapy in Dementia-UK intervention. *BMC Geriatrics* 2019;19(1):364. doi: 10.1186/s12877-019-1385-7

For peer review only

11. Mountain G, Windle G, Hind D, et al. A preventative lifestyle intervention for older adults (lifestyle matters): a randomised controlled trial. *Age and Ageing* 2017;46(4):627-34. doi: 10.1093/ageing/afx021

12. Clark F, Azen S, Zemke R, et al. Occupational Therapy for Independent-Living Older Adults. *Journal of the American Medical Association* 1997;278(16):1321-6.

13. Clark F, Jackson J, Carlson M, et al. Effectiveness of a lifestyle intervention in promoting the well-being of independently living older people: results of the Well Elderly 2 Randomised Controlled Trial. *Journal of Epidemiology & Community Health* 2011

14. Mountain G, Sprange K, Chatters R. Lifestyle Matters Randomized Controlled Trial of a Preventive Health Intervention for Older People: Qualitative Sub Study with Participants and Intervention Facilitators. *Clinical interventions in aging* 2020;15:239-53. doi: 10.2147/cia.s232108 [published Online First: 2020/02/29]

15. Moncher F, Prinz R. Treatment fidelity in outcome studies. *Clinical Psychology Review* 1991;11:247-66.

16. Borrelli B. The assessment, monitoring, and enhancement of treatment fidelity in public health clinical trials. *Journal of Public Health Dentistry* 2011;71:S52-S63.

17. Medical Research Council (MRC). A FRAMEWORK FOR DEVELOPMENT AND EVALUATION OF RCTs FOR COMPLEX INTERVENTIONS TO IMPROVE HEALTH, 2000.

18. Medical Research Council (MRC). Developing and evaluating complex interventions 2019.

19. Bandura. A. Self-efficacy: The exercise of control.: New York: Freeman 1997.

20. Luszczynska A, Gutierrez-Dona B, Schwarzer R. General self-efficacy in various domains of human functioning: Evidence from five countries. *International Journal of Psychology* 2005;40(2):80-89.

21. Bandura A. Self-efficacy mechanism in human agency. *American Psychologist* 1982;37(2):122-47. doi: 10.1037/0003-066X.37.2.122

22. Law M, Cooper B, Strong S, et al. The Person-Environment-Occupation Model: A Transactive Approach to Occupational Performance. *Canadian Journal of Occupational Therapy* 1996;63(1):9-23. doi: 10.1177/000841749606300103

For peer review only

23. Hasson H, Blomberg S, Duner A. Fidelity and moderating factors in complex interventions: a case study of a continuum of care program for frail elderly people in health and social care. *Implement Sci* 2012;7:23. doi: 10.1186/1748-5908-7-23 [published Online First: 2012/03/23]

24. Medical Research Council. Developing and evaluating complex interventions: new guidance. London: MRC; 2008

25. Toomey E, Hardeman W, Hankonen N, et al. Focusing on fidelity: narrative review and recommendations for improving intervention fidelity within trials of health behaviour change interventions. *Health Psychology and Behavioral Medicine* 2020;8(1):132-51. doi: 10.1080/21642850.2020.1738935

26. Walton H, Spector A, Tombor I, et al. Measures of fidelity of delivery of, and engagement with, complex, face-to-face health behaviour change interventions: A systematic review of measure quality. *British journal of health psychology* 2017;22(4):872-903. doi: 10.1111/bjhp.12260 [published Online First: 2017/08/02]

27. Wright J, Foster A, Cooper C, et al. Study protocol for a randomised controlled trial assessing the clinical and cost-effectiveness of the Journeying through Dementia (JtD) intervention compared to usual care. *BMJ Open* 2019;9(9):e029207. doi: 10.1136/bmjopen-2019-029207

28. Brooke P, Bullock R. Validation of a 6 item cognitive impairment test with a view to primary care usage. *International journal of geriatric psychiatry* 1999;14:936-40.

29. Sprange K, Mountain GA, Brazier J, et al. Lifestyle Matters for maintenance of health and wellbeing in people aged 65 years and over: study protocol for a randomised controlled trial. *Trials* 2013;14:302-02. doi: 10.1186/1745-6215-14-302

30. Carroll C, Patterson M, Wood S, et al. A conceptual framework for implementation fidelity. *Implement Sci* 2007;2:40. doi: 10.1186/1748-5908-2-40 [published Online First: 2007/12/07]

31. Craig C, Mountain G. Lifestyle Matters: an occpational approach to healthy ageing: Bicester: Speechmark 2007.

32. Hawe P, Shiell A, Riley T. Complex interventions: how “out of control” can a randomised controlled trial be? *BMJ* 2004;328(7455):1561-63. doi: 10.1136/bmj.328.7455.1561

For peer review only

33. National Institute for Health and Clinical Excellence (NICE). Public Guidance 6. Behaviour change: general approaches 2007 [Available from: http://guidance.nice.org.uk/PH6/Guidance/pdf/English.

34. Mountain G, Mozley C, Craig C, et al. Occupational therapy led health promotion for older people: feasibility of the lifestyle matters programme. *British Journal of Occupational Therapy* 2008;71(10):406-13.

35. Pope C, Mays N. Reaching the parts other methods cannot reach: an introduction to qualitative methods in health and health services research. *BMJ : British Medical Journal* 1995;311(6996):42-45.

36. Masterson-Algar P, Burton CR, Rycroft-Malone J, et al. Towards a programme theory for fidelity in the evaluation of complex interventions. *J Eval Clin Pract* 2014;20(4):445-52. doi: 10.1111/jep.12174 [published Online First: 2014/05/21]

37. McHugh ML. Interrater reliability: the kappa statistic. *Biochem Med (Zagreb)* 2012;22(3):276-82. [published Online First: 2012/10/25]

38. Gale N, Heath G, Cameron E, et al. Using the framework method for the analysis of qualitative data in multi-disciplinary health research. *BMC Med Res Methodol* 2013;13(117)

39. Ritchie J, Spencer L. Qualitative data analysis for applied policy research: The Qualitative Researcher's Companion: SAGE Publications 2002.

40. DG A. Practical statistics for medical research: London: Chapman and Hall 1991.

41. Mars T, Ellard D, Carnes D, et al. Fidelity in complex behaviour change interventions: a standardised approach to evaluate intervention integrity. *BMJ open* 2013;3(11):e003555. doi: 10.1136/bmjopen-2013-003555 [published Online First: 2013/11/19]

42. Pérez D, Van der Stuyft P, Zabala MdC, et al. A modified theoretical framework to assess implementation fidelity of adaptive public health interventions. *Implementation Science* 2016;11(1):91. doi: 10.1186/s13012-016-0457-8

43. Proctor E, Silmere H, Raghavan R, et al. Outcomes for implementation research: conceptual distinctions, measurement challenges, and research agenda. *Administration and policy in mental health* 2011;38(2):65-76. doi: 10.1007/s10488-010-0319-7 [published Online First: 2010/10/20]

For peer review only - http://bmjopen.bmj.com/site/about/guidelines.xhtml

44. Duncan EAS, Nicol MM. Subtle Realism and Occupational Therapy: An Alternative Approach to Knowledge Generation and Evaluation. *The British Journal of Occupational Therapy* 2004;67(10):453-56. doi: 10.1177/030802260406701006

45. Carroll KM, Nich C, Sifry RL, et al. A general system for evaluating therapist adherence and competence in psychotherapy research in the addictions. *Drug and alcohol dependence* 2000;57(3):225-38. doi: 10.1016/s0376-8716(99)00049-6 [published Online First: 2000/02/08]

46. Song M, Happ M, Sandelowski M. Development of a tool to assess fidelity to a psycho-educational intervention. *J Adv Nurs* 2010;66(3):673-82.

47. Resnick B, Inguito P, Orwig D, et al. Treatment Fidelity in Behaviour Change Research: a case example. *Nursing Research* 2005;54(2):139-43.

48. Moore G, Audrey S, Barker M, et al. Process evaluation in complex public health intervention studies: the need for guidance. *J Epidemiol Community Health* 2014;68(2):101-2. doi: 10.1136/jech-2013-202869 [published Online First: 2013/09/12]

49. Hoddinott P, Allan K, Avenell A, et al. Group interventions to improve health outcomes: a framework for their design and delivery. *BMC Public Health* 2010;10(800)

For peer review only

Lifestyle Matters Additional File 1

Contents

Document 1: Lifestyle Matters training delivery observation checklist 2

Document 2: Lifestyle Matters intervention delivery video checklist 3

For peer review only

Document 1: Lifestyle Matters training delivery observation checklist

**TRAINER**	
**Domain**	**Criteria**
Trainer	1. The trainer is the same for all training
	2. The trainer is an author of the programme
	3. Demonstrates a range of group work skills (didactic teaching, peer sharing, interactive activities, active experimentation etc)
Materials	4. PowerPoint presentations and materials directly relate to the goals of the training
	5. Provides a CD of training content and materials to all trainees
Tailoring	6. Training is tailored to recipients' skill level, experience or work background
	7. Training is tailored in response to the observed needs of the trainees, and feedback from the trainees during the sessions
Content	8. The goals of the training are covered
	9. Examples of the core themes and examples of subsidiary themes are covered
	10. Mandatory themes are covered:<br>• A celebration of achievements<br>• Activity and health<br>• Endings
**TRAINEES**	(Performance in discussion, role play, problem solving, expressive exercises, reflective exercises shared, written tests)
**Domain**	**Criteria**
Understanding	11. Critically discuss demographic changes and the concept of active ageing
	12. Discuss the evidence for Lifestyle matters
	13. Identify the ideas that are key to Lifestyle Matters, including<br>• enabling group members to do things for themselves<br>• transferring skills from the safety of the group to their wider lives
	14. Discuss the content of the Lifestyle Matters manual and how to apply it
Self -awareness	15. Share their prior expertise, hopes and expectations of the training
	16. Share their own perceptions of ageing and how this may impact on the programme
	17. Reflect on and share their own facilitation style and skills
Skill acquisition	18. Use the LifeStyle Matters manual to practice planning sessions within themes
	19. Identify how to locate and access community resources
	20. Respond to potential scenarios, using creative problem solving and considering individual needs (including risk taking)
Group work	21. Understand and demonstrate a range of group work skills (didactic teaching, peer sharing, interactive activities, active experimentation etc)
	22. Understand and demonstrate how to manage group dynamics
	23. Understand and demonstrate an enabling style of group facilitation
	24. Demonstrate how to tailor the intervention to the needs and choices of group members
Individual sessions	25. Explore the value of 1:1 sessions and potential activities to use in these
Group outings	26. Explore the value or outings and how to help group members to plan them
Reflection & evaluation	27. Explore reflective practice and discuss its value
	28. Practice evaluating and planning actions
Supervision	29. Discuss the value and principles of supervision

For peer review only

Document 2: Lifestyle Matters intervention delivery video checklist

FACILITATOR	
**Domain**	**Criteria**
Resources	30. Two facilitators deliver
Personal qualities	31. Shows warmth and empathy
	32. Listens actively and respectfully
Enabling	33. builds on the assets, skills and abilities that each person brings
	34. Encourages the group to make choices
Group work skills	35. Facilitates communication and interaction (to ground rules?)
	36. Draws in quiet members of the group
	37. Facilitates peer exchange and guidance, and peer modelling
	38. Manages space, time and opportunity effectively
	39. Facilitates the members to explore differences between them, or conflict and resolution
	40. Builds mutual support and group cohesion
Goals and needs	41. Balances meeting individual needs and goals and group needs
	42. Balances sufficient challenge in activities with ensuring they are achievable
	43. Tailors activities to the needs of members
Content	44. Focuses on the themes in the programme, or new themes chosen by group members
	45. Uses group activities, not just discussion (activities from the manual or from elsewhere)
	46. With members, selects and plans group outings that explore and extend the themes in the programme
**GROUP MEMBERS**	
**Domain**	**Criteria**
Understanding	47. The relationship between activity, health and aging, or one of the other themes from the programme
Occupational self-analysis	48. Analyse their own daily occupations (their significance, impact on health, components) and make choices
	49. Sharing and reflecting on experiences of participating in activities (Occupational storytelling)
Goal setting	50. Own goals identified within the group and within activities
Grading and layering	51. Grading and layering of activities to constantly revisit and extend skills and build confidence
Mutual support	52. Show mutual appreciation and support
Creativity	53. Practice any of: self-expression, innovation, problem solving and adaptation within the safety of the group
	54. Engage in enjoyable and stimulating activities
Older people as experts	55. Acknowledge and appreciate their own skills and how they can contribute to the group
	56. Bring in and share their own skills and knowledge
	57. Group members in turn take leadership or contribute ideas
Transfer to everyday life	58. build skills, solutions or confidence within the group
	59. Report the transferring of skills, solutions or confidence from the group to wider life
Change over time	60. Plans for individuals are taken forward, whilst respecting each person's need to balance opportunities and risk
Confidence and hope	61. Achievements are celebrated
	62. Adventures and dreams are welcomed

For peer review only

BMJ Open

Evaluation of intervention fidelity in a randomised controlled trial of a complex psychosocial intervention: Lifestyle Matters

Journal:	*BMJ Open*
Manuscript ID	bmjopen-2020-043478.R2
Article Type:	Original research
Date Submitted by the Author:	22-Mar-2021
Complete List of Authors:	Sprange, Kirsty; University of Nottingham, Nottingham Clinical Trials Research Unit, University Park, NG7 2RD<br>Mountain, Gail; University of Bradford, Centre for Applied Dementia Studies, Faculty of Health Studies, Richmond Road, Bradford, BD7 1DP<br>Craig, Claire; Sheffield Hallam University, Lab4Living, Furnival Building, 153 Arundel Street, Sheffield, S1 2NU
**Primary Subject Heading**:	Research methods
Secondary Subject Heading:	Geriatric medicine
Keywords:	Dementia < NEUROLOGY, GERIATRIC MEDICINE, Clinical trials < THERAPEUTICS

SCHOLARONE™
Manuscripts

I, the Submitting Author has the right to grant and does grant on behalf of all authors of the Work (as defined in the below author licence), an exclusive licence and/or a non-exclusive licence for contributions from authors who are: i) UK Crown employees; ii) where BMJ has agreed a CC-BY licence shall apply, and/or iii) in accordance with the terms applicable for US Federal Government officers or employees acting as part of their official duties; on a worldwide, perpetual, irrevocable, royalty-free basis to BMJ Publishing Group Ltd ("BMJ") its licensees and where the relevant Journal is co-owned by BMJ to the co-owners of the Journal, to publish the Work in this journal and any other BMJ products and to exploit all rights, as set out in our licence.

The Submitting Author accepts and understands that any supply made under these terms is made by BMJ to the Submitting Author unless you are acting as an employee on behalf of your employer or a postgraduate student of an affiliated institution which is paying any applicable article publishing charge ("APC") for Open Access articles. Where the Submitting Author wishes to make the Work available on an Open Access basis (and intends to pay the relevant APC), the terms of reuse of such Open Access shall be governed by a Creative Commons licence – details of these licences and which Creative Commons licence will apply to this Work are set out in our licence referred to above.

Other than as permitted in any relevant BMJ Author's Self Archiving Policies, I confirm this Work has not been accepted for publication elsewhere, is not being considered for publication elsewhere and does not duplicate material already published. I confirm all authors consent to publication of this Work and authorise the granting of this licence.

For peer review only

Evaluation of intervention fidelity in a randomised controlled trial of a complex psychosocial intervention: Lifestyle Matters

Authors

Kirsty Sprange, University of Nottingham, Nottingham Clinical Trials Unit, University Park, NG7 2RD, UK. kirsty.sprange@nottingham.ac.uk (Corresponding author) ORCiD 0000-0001-6443-7242

Gail A Mountain University of Bradford, Centre for Applied Dementia Research, Faculty of Health Studies, Richmond Road, Bradford, BD7 1DP, UK g.mountain@bradford.ac.uk ORCiD 0000-0002-5417-7691

Claire Craig, Sheffield Hallam University, Lab4Living, Furnival Building, 153 Arundel Street, Sheffield, S1 2NU, c.craig@shu.ac.uk ORCiD 0000-0002-3475-3292

For peer review only

Abstract

Objectives: Robust research of complex interventions designed to promote mental wellbeing in later life is required to inform service development. An essential component is ensuring that such interventions are delivered as intended. We present a detailed description of the design and implementation of a fidelity assessment within a trial of one such intervention (Lifestyle Matters). The findings help to explain the trial results and also inform the design of embedded fidelity assessments within future evaluations of complex interventions.

Design: We conducted a mixed method fidelity assessment embedded as part of a multi-centre pragmatic randomised controlled trial. A conceptual fidelity framework was developed from the Behavior Change Consortium framework. From this the fidelity assessment was designed. The resulting instrument assessed the following parameters: intervention design, training, supervision; and delivery, receipt and enactment of the intervention.

Intervention: The Lifestyle Matters intervention was designed to assist older people to improve and sustain mental wellbeing through participation in meaningful activity. The aim is to enable participants to engage in both new and neglected activities through a mix of facilitated group meetings and individual sessions.

Results: The fidelity assessment demonstrated that the intervention was delivered as per protocol for the group component and was tailored to meet individual needs. There was substantial inter-rater agreement for training; and group member performance 0.72; and moderate agreement for facilitator performance 0.55. It was not possible to determine whether small declines seen in facilitator performance were due to facilitator drift or moderating factors such as group dynamics or participant characteristics.

Conclusions: The assessment methods adequately measured criteria identified as being significant indicators of fidelity. Adherence during training, delivery and supervision was good. The subjective nature of identification and rating observed behaviours was the main challenge. Future research should explore alternative methods of assessing fidelity in trials of complex interventions.

Trial registration: ISRCTN67209155

For peer review only

Keywords: Fidelity assessment, complex intervention, dementia

Strengths and Limitations of this study

- This study demonstrated that it is feasible to design a fidelity assessment framework and develop bespoke assessment tools to evaluate a complex multi-component psychosocial intervention.
- The extent of the assessment was limited by the methods used, for example it was not appropriate or even ethical to undertake observations of activities in the community.
- The validity of the findings is reliant on whether bespoke assessment tools are sensitive enough to identify the complex constructs within this type of intervention.

Background

An increase in long term conditions within the global population combined with increased longevity has led to interest in how tailored psychosocial interventions might enable individuals to retain physical and mental wellbeing in the extended lifespan[1 2]. UK guidance has identified the need for multi-component, tailored individual and group based preventative interventions which promote health and wellbeing through occupation and physical activity[3]. The benefits of social networking and group interventions involving activity targeting social isolation and loneliness has also been identified[4] but there is a limited range of recommended interventions. The challenges of associated research include intervention development and evaluation of intervention acceptability, followed by robust testing to determine intervention effectiveness[3]. Robust testing should include an assessment of intervention fidelity to assess the extent to which the intervention is delivered as intended.

Fidelity assessment and maintenance of fidelity[5] enables researchers to demonstrate with greater confidence that study results were due to the intervention and not to other confounding factors. It is acknowledged that using multiple methods as part of a fidelity assessment can enhance validity and reliability of complex interventions[6]. Development and application of such multi-method fidelity assessments in complex multi-component interventions have been demonstrated as feasible[7-9].

A pragmatic randomised controlled trial (RCT) with an embedded fidelity assessment was conducted to evaluate a complex manualised intervention, Lifestyle Matters, which was created to promote the mental wellbeing of community living older people[10]. Lifestyle

For peer review only - http://bmjopen.bmj.com/site/about/guidelines.xhtml

Matters was developed for the UK setting from a US intervention, Lifestyle Redesign which was found to promote physical and mental health and wellbeing, occupational functioning and life satisfaction in older adults[11 12]. As reported in [10], the trial fully adhered to Consort guidelines. There is some debate about the benefits or otherwise of the manualisation of complex interventions but one argument suggests that manaualisation can reduce variance in intervention delivery thereby enhancing fidelity[13].

Fidelity assessment for the Lifestyle Matters study included an evaluation of facilitator training and intervention delivery against pre-determined criteria; and a complementary qualitative exploration of the views of facilitators regarding how lifestyle change might be supported or not through the intervention and their views of how they had been able to deliver this goal[14].

..

MRC guidance recommends that complex intervention design and associated fidelity assessment frameworks are developed from the underlying intervention theory[15]. Lifestyle Matters is underpinned by social cognitive theory. There is a focus upon promotion of self-management by participants[16], and in particular upon developing and sustaining self-efficacy as this is known to be a protective factor in improving motivation and emotional wellbeing[17]. Intervention theory is located in the promotion of self-efficacy[18] as well as a model that proposes that the transactional relationships between the person, environment and activities influence occupational performance. Occupation in this context refers to meaningful activity and active participation in life[19]. The intervention guides participants to identify their own personal goals and work towards them. They are encouraged to use peer support through the group and support from facilitators in both group and individual sessions to actively overcome barriers to meaningful participation within their communities.

Interventions like Lifestyle Matters will have core components such as number of group and individual sessions and structured manual topics. However adapting a complex intervention to meet the needs of individual participants is important[15 20]. The Lifestyle Matters intervention provides a content and delivery structure, which is both flexible and readily tailored to the needs of participants. Therefore, a range of methods and tools to assess and evaluate the fidelity of this tailored intervention were required. This was important as the application of measures to assess intervention adaptation can improve our understanding of how different components can impact upon delivery and receipt in context[5 21].

For peer review only - http://bmjopen.bmj.com/site/about/guidelines.xhtml

This paper describes the fidelity assessment conducted for the Lifestyle Matters study and presents the findings from analysis of facilitator training and supervision, intervention delivery and receipt. The findings are then discussed in the context of intervention implementation.

Trial design

The study was a pragmatic, two-arm, parallel group, individually randomised controlled trial designed to determine the population benefit of an occupation based intervention for people aged 65 years or older[10]. A total of 288 participants were randomised between 14 August 2012 and 19 April 2013 (18 couples and 252 individuals); 145 and 143 were allocated to the intervention and control groups, respectively. The intervention was delivered in one city in the north of England and in North Wales. Participants were eligible if they had reasonable cognitive function to participate in the intervention as evidenced by a score of 0 to 7 on the Six Item Cognitive Impairment Test (6CIT)[22]. They were also living independently or in sheltered accommodation, alone or with others and able to converse in English or Welsh. An associated fidelity assessment and qualitative sub-study were conducted to evaluate facilitator training and supervision; intervention delivery; and intended changes to lifestyle by participants. The latter are reported elsewhere[14 23].

The Lifestyle Matters intervention

The intervention involves volunteers taking part in sixteen facilitated sequential weekly group meetings with up to 15 others and in four individual sessions with one of the facilitators to pursue individual goals[10 24]. During group meetings participants are encouraged to think about and engage in discussion and activities to explore the relationship between occupation and health, selecting from a menu of potential topics. A crucial element is going into the community to try out new or neglected skills with support from each other and from the facilitators; at least four sessions should involve such activities.

Group meetings are approximately two hours in duration at a convenient community venue. The first group sessions are structured to cover key topics from the manualised intervention. This provides a foundation for facilitators to maintain adherence to the intervention and for

For peer review only - http://bmjopen.bmj.com/site/about/guidelines.xhtml

both facilitators and participants to work together on future sessions using the same model. Short breaks of no more than two weeks can be included to accommodate statutory holidays.

Individual sessions are an important intervention component and are specifically directed by the individual's goals and preferences. The first session concentrates on the participant-facilitator relationship. The remaining three sessions are built around the participant and what they want to achieve, using the intervention to assist them to execute this.

Facilitators and facilitation

Facilitators with the necessary skill set and qualities were recruited to deliver the intervention as intended[25]. A job description was produced which detailed core requirements, deemed to be essential for understanding the intervention and enabling successful delivery. These included prior experience of working with groups and with older people in community settings and specifically the planning and delivery of group and individual sessions. A requirement of intervention delivery is for two facilitators to be present during each group and to share working with participants during individual sessions. Facilitators could be involved in the delivery of up to three intervention groups of 8-16 participants at any one time (including associated individual sessions).

Facilitator Supervision

Supervision was provided by appropriately experienced Occupational Therapists who also attended facilitator training alongside the facilitators. A protocol was created to enable provision of consistent and appropriate supervision across and within sites. Regular one-to-one supervision was recommended on a weekly basis at a mutually convenient time and place. Preferably face-to-face but with distance supervision being an option if appropriate. Joint supervision was also deemed acceptable if the individual supervisory needs of facilitators had been met.

Training

A two day intensive training course was delivered jointly to all facilitators and supervisors before intervention delivery commenced, thereby ensuring that everyone received the same information and training experience to equip them to deliver the intervention and improve fidelity[26].

For peer review only - http://bmjopen.bmj.com/site/about/guidelines.xhtml

Training content included presenting the rationale and theory behind the intervention and intervention components[25]. The training then mirrored delivery of Lifestyle Matters intervention and manual[27] in that it involved;

- understanding the importance of taking a personalised and tailored approach towards participants at all times[28].
- didactic sessions to explain the programme and its ethos.
- peer sharing; providing the opportunity to share narratives, explore experience and attitudes and reflect on the relationship that can develop between participants and between facilitators and participants and between supervisor and facilitators.
- undertaking active experimentation, thereby testing out ideas and concepts.
- exploring communication skills through role playing, vignettes and group discussions.

The two-day training was video recorded to give facilitators an idea of how they and their participants might experience this for fidelity assessment and smooth out any challenges with using the recording equipment.

All facilitators and supervisors received a copy of the manual and an accompanying CD-Rom with downloadable resources for intervention delivery[27].

Materials and methods

Fidelity assessment framework

A comprehensive fidelity assessment framework specific to the intervention was developed prior to intervention delivery (see Table 1) based on the Behaviour Change Consortium (BCC) recommendations and NICE guidance on behaviour change[29 30]. Fidelity assessment and quality assurance parameters centred on the BCC five recommended criteria: intervention design, training, delivery, receipt and enactment. For each criterion, programme objectives based on the Lifestyle Matters manualised intervention and training were discussed and agreed by two senior researchers. A description for each standard was then developed and methods to demonstrate fidelity to each standard identified (see Table 1).

Table 1: Lifestyle Matters fidelity assessment framework and results

Element	Fidelity criteria	Fidelity standard	Standard met	Evidence
**Trial Design**				
Comparable treatment	All participants received the	• 16 weekly meetings offered to all participants. Attendance recorded.	Yes	• Trial documentation e.g. Information sheet.<br>• 176 (11x16) weekly registers recorded.

For peer review only - http://bmjopen.bmj.com/site/about/guidelines.xhtml

	same programme tailored to the needs of the group/ setting.	• 4 individual sessions offered to all participants. Attendance recorded.<br>• 4 out of venue sessions offered to all participants. Attendance recorded.	Yes	• Trial documentation e.g. Information sheet.<br>• 443 individual sessions offered, 143 taken-up (32%).<br>• On average participants took part in four out of venue activities across the 16 weeks.
Risk to implementation	Plan for potential issues that could affect the delivery of the lifestyle matters programme.	• A range of recruitment strategies will be implemented.	Yes	• Methods used included GP mail-outs, direct referrers and posters/leaflets.
		• A range of days and times for meetings offered, from which participants can choose.	Yes	• Facilitators worked flexibly.<br>• Groups took place on various days of the week and times of day (AM/PM) .
		• Undertake three recruitment cycles in three geographical areas, to prevent saturation.	Yes	• Recruitment conducted in multiple areas at each site.
**Monitoring provider training**				
Standardised training and facilitator skill acquisition	All facilitators receive the same training tailored to the group/setting and engage with the training in a similar way.	• Completion of training exercise by facilitators.	Yes	• Observation by researchers of participants completing role playing exercises and group work.
		• Training delivered by the same trainer.	Yes	• Same trainer delivered all training.
		• Manual/CD-Rom provided to all trainees.	Yes	• Manual (or copy) and CD provided to all trainees.
		• Observation of the training session by two researchers using a content checklist	Yes	• Observation by two researchers of skill transference using content checklist.<br>• Checklist coding developed to observe 'All' participants.
**Monitoring intervention delivery**				
Standardised delivery	All facilitators using the same techniques and content from the programme.	• Observation using a content checklist by two researchers	Yes	• Observation completed by two researchers.
		• 75% of opportunities for completing goal setting are recorded (for individual and group).	No	• Participants found concept of 'goal setting' difficult to understand. 'Goal setting' sheets provided in programme rarely used. Discussion of 'Planning' and 'Wishes' preferred.
		• Range of materials from the Lifestyle Matters programme received by all participants.	Yes	• Observation of manual materials used during recorded group meetings.<br>• Use of materials discussed by participants during qualitative interviews.
		• Facilitators maintain reflective diaries.	Yes	• Used as part of supervision sessions, confirmed by facilitators and supervisors.
		• Registers completed for weekly meetings and individual sessions.	Yes	• Registers completed and recorded on the trial database.
		• Participant and facilitator semi-structured interview topic guide.	Yes	• Interviews conducted with all facilitators at the end of cycle 1 and cycle 3. Interviews conducted with 10% sample of participants at the end of their programme attendance.
		• All participants receive certificate of attendance/achievement.	Yes	• Copies of certificates given to all participants across both sites.
		• Facilitators meet the NHS Band 4 equivalent job description	Yes	• Facilitators selected for interview based on meeting essential criteria of job description.
Minimise drift in skills/ delivery	Adherence to training content and delivery over the 3 cycles of the intervention	• Observation using a content checklist by two researchers	Yes	• Observation by two observers.
		• OT supervisor protocol.	Yes	• Protocol written and provided to supervisors and facilitators prior to the programme start.
		• Each facilitator will attend between 8 and 16 supervision sessions in total of which half should be delivered face-to face.	Yes	• Supervisor attendance records provided for two facilitators (consent obtained).<br>• Interviews – Reported face-to-face contact recorded for one facilitator for 12 months. Three facilitator's supervision started as face-to-face moving to a combination with telephone contact.
**Monitoring receipt of intervention**				
Participant attendance and engagement	Recording participants weekly attendance	• Registers completed by facilitator for weekly meetings and individual sessions where arranged.	Yes	• All registers completed and recorded.<br>• Interviews demonstrated that most participants took part in the programme, although not all joined every activity mainly due to health or mobility issues.
	All participants taking part in the meetings and activities	• 75% of opportunities for completing goal setting are recorded (individual and group).	No	• Concept of 'goal setting' difficult to understand by participants. 'Planning' or 'Wishes' preferred.
		• Participant and facilitator semi-structured interview topic guide.	Yes	• All Facilitators interviewed at cycle 1 and cycle 3.

For peer review only - http://bmjopen.bmj.com/site/about/guidelines.xhtml

	Impact of intervention on participant in terms of wellbeing			• 10% sample of participants interviewed.<br>• Participant behaviours observed during videoed sessions.
		• PROMS.	Awaiting results	• Completed PROMS recorded on trial database.

Adapted from Bellg et al, 2004[29]

Assessment tools

The underlying ethos of Lifestyle Matters and the tailored approach to delivery make fidelity assessment complex as many of the identified criteria are subjective. A range of quantitative and qualitative tools were therefore used in line with MRC recommendations for complex interventions[15], including attendance registers, observation checklists and interview schedules which were all piloted before application.

Intervention attendance registers

Registers were used to routinely record both group and individual session attendance. Using pilot study findings it was estimated that participants would need to attend at least half (eight) group meetings, to benefit from the intervention[31]. The number of individual sessions required to derive benefit was not set.

Development of observation checklists

Observation checklists were developed by two senior researchers who identified domains comprising the core skills and key criteria identified in the Lifestyle Matters manual, worksheets and training materials (see additional file 1). Criteria described the components and behaviours expected to be present during intervention delivery. Domains were divided into those associated with 'Trainer or Trainee performance' and those associated with 'Facilitator or Group member performance'.

A scoring system was devised. Several criteria were rated on a binary 'Yes' or 'No' scale for presence or absence. For the purpose of analysis if a criterion was present this was converted to a score of 3. If the criterion was not present this was converted to a score of 0.

Fidelity scores were calculated based on agreement of a final score between the two coders and the total was given a percentage score as follows. The scoring system was adapted from the principle set out in Borelli 2011, that 80-100% constitutes high fidelity[25]:

- 0- 60% Unsatisfactory

For peer review only

- 61-70% Satisfactory
- 71-80% Good
- 81-90% Very good
- 91-100% Excellent

To ensure consistency the same two researchers completed fidelity assessment activities and coding. The patient and public contributor for the project also contributed to the fidelity assessment analysis including reviewing and piloting observation checklists (as described below), as well as participant observation during training.

Assessment of the fidelity of training delivery

Participant observation, in which the researcher not only observes, but takes an active role in the setting[32], was selected to assess the fidelity of training delivery. The researchers therefore attended the training as delegates. This decision was taken as participant observation allows the observer to not only have the same experience as those taking part, as an insider, but also enables them understand the more subtle aspects of delivery such as group dynamics as an outsider[33]. However, this can also result in loss of perspective and objectivity to some extent.

During the training, sessions were independently rated by the two researchers. There were also discussions between the trainer and researchers at the beginning and end of each day to review content and feedback from attendees. All scores were then shared and reviewed for reliability of interpretation and coding against checklist criteria.

Assessment of the fidelity of intervention delivery

A purposive sample of eight video-recordings were taken of four of the 11 Lifestyle Matters groups to monitor implementation and adherence to the intervention and identify any facilitator drift over time. To reduce bias the same number of groups and group meetings from both sites were selected for assessment. Written consent to conduct video recordings was obtained from each participant and facilitators recorded the group meetings using simple compact video recorders.

One video of each of the four selected groups was recorded at approximately week four and again at around week 10 of intervention delivery although some flexibility in session choice was necessary to avoid out of venue activities (see Table 2). Observing two meetings per

For peer review only - http://bmjopen.bmj.com/site/about/guidelines.xhtml

group; one at the beginning and one near the end enabled us to examine any learning effects and identify any facilitator drift[34].

Table 2: Sample of video recorded group meetings

Site	Wave	Lifestyle Matters Group	Programme Week (1-16) videoed	No of Facilitators present	Participants attending/ registered
North England	1	1	6	2	10/13 (77%)
			10	2	11/13 (85%)
North Wales	1	1	4	2	7/10 (70%)
			10	2	7/9† (78%)
North England	3	6*	7	2	6/8 (75%)
			15	2	7/8 (88%)
North Wales	3	5*	4	2	8/9 (89%)
			10	2	7/9 (78%)

*North England site completed six Lifestyle Matters groups and North Wales completed five

†Participant withdrew

Video content was then rated using the predetermined checklist and findings analysed to determine delivery and receipt of the intervention and any change over time.

During piloting of the intervention fidelity checklist, it became apparent that several of the assessment criteria could not be observed during the weekly group meetings. For example, receipt and enactment of the intervention would occur in the participant's everyday life away from the group. It was deemed that either the criterion could not be present during every weekly meeting or it would be necessary to observe several meetings consecutively which was not planned. Further observations using the checklist to test the assumption found this to be consistent. The criteria deemed to be immeasurable were therefore excluded from the final analysis.

Due to practicalities, only the groups held within the meeting venue were observed. Given the importance of enactment of activities in the community this was a limitation.

We did not undertake observations of the individual sessions as these were deemed a safe space for participants to work with their facilitator on individual goals. These sessions could also involve activities in the community where it would be impractical and unethical for researchers to observe sessions.

For peer review only - http://bmjopen.bmj.com/site/about/guidelines.xhtml

Inter-rater reliability

Inter-rater reliability demonstrates whether available data accurately represents the variables being measured and the extent to which coders attribute the same score to the same variable[35]. Inter-rater reliability was calculated by two researchers independently coding video recordings and by scores being compared using the Kappa statistic, with a value of 0.61 and above indicating good inter-rater agreement.

- < 0.20 Poor
- 0.21-0.40 Fair
- 0.41-0.60 Moderate
- 0.61-0.80 Good
- 0.81-1.00 Very good

Semi-structured interviews

Semi-structured interviews were undertaken with all facilitators to explore views on what enables effective intervention facilitation, and the skills and competencies required to deliver the intervention and enable participants to act on it[14 23]. Interviews took place at the end of the first and third (final) wave of intervention delivery to identify any changes in experiences of delivery over time.

An interview topic guide was developed informed by the content of the training materials and intervention manual as well as knowledge gained from prior feasibility work[31].

A purposeful sample of 10% of participants were interviewed; selected from both sites and from each of the three waves of intervention delivery to elicit the range and nature of issues that influenced their experiences of the intervention. The sample was considered adequate to reach data saturation within resource limitations[36].

All transcripts were read for familiarisation and annotated for themes by the same two researchers (Fidelity lead and second researcher) using Framework analysis in NVivo[37 38]. For the purposes of the fidelity assessment, data from the interviews were used to identify cross cutting themes and explanatory factors for fidelity outcomes.

For peer review only - http://bmjopen.bmj.com/site/about/guidelines.xhtml

Patient and public involvement

Patients and members of the public were involved at all stages from the development of the intervention during feasibility testing through to trial design and conduct. We received input from older adults on the design of the trial materials and in the oversight of the delivery of the study through PPI representation on trial oversight groups. The main trial results were disseminated to participants using materials suitable for a non-specialist audience and shared with the wider public, clinical and academic colleagues through events and publications.

Results

The 'Fidelity Assessment Framework' including evidence for fidelity is presented in table 1.

Facilitators

All four facilitators remained the same throughout the trial at both sites. They were all female, younger than the youngest participant (65 years) and from a health or social care professional background.

Intervention attendance

Eleven Lifestyle Matters interventions were delivered, six in one city in Northern England and five in North Wales. Four groups were selected for the fidelity assessment as previously described. Overall attendance at the observed group sessions was 70% or above (see table 2). The average number of out of venue activities conducted as a part of the group aspect of the intervention across all groups was four.

Training delivery

The two-day training was attended by all four facilitators (two from each site) and the two supervisors (one from each site).

Excellent fidelity was demonstrated for trainer performance with a score of 100% across all four domains.

Good fidelity was identified through a criterion score of 75.4% across eight domains for trainees. Overall training fidelity was very good with a total agreed criterion score for ‘trainer performance’ and ‘trainee performance’ of 83.9%.

For peer review only - http://bmjopen.bmj.com/site/about/guidelines.xhtml

Intervention delivery

Facilitator performance

Excellent or very good fidelity was achieved in four out of the eight recorded groups (see Table 3). Only one observed group (Wave 1, Site A, Video 2) demonstrated unsatisfactory fidelity (52%). The other three groups all showed either an increase or maintenance in fidelity scores.

Table 3: Fidelity scores for 'Facilitator performance'

	**Facilitator score /out of 48**							
**Wave 1**	**(Site A) Group 1**				**(Site B) Group 1**			
	**Rater 1**	**Rater 2**	**Agreed score**	**%**	**Rater 1**	**Rater 2**	**Agreed score**	**%**
Video 1	38	33	37/48	**77.0%**	38	39	39/48	**81.2%**
Video 2	22	28	25/48	**52.0%**	44	44	42/48	**87.5%**
**Wave 3**	**(Site A) Group 6**				**(Site B) Group 5**			
	**Rater 1**	**Rater 2**	**Agreed score**	**%**	**Rater 1**	**Rater 2**	**Agreed score**	**%**
Video 1	44	44	44/48	**91.6%**	30	34	30/48	**62.5%**
Video 2	46	45	44/48	**91.6%**	33	35	34/48	**70.8%**

Excellent or very good fidelity was demonstrated in three of the six assessment domains. Only one domain; 'Goals and Needs' was rated as being unsatisfactory. This required evidence of programme tailoring and how facilitators helped participants to select achievable activities with appropriate levels of challenge.

The decline in fidelity seen at Site A during Wave 1 (77% to 52%), could be explained by the facilitators arranging for two external speakers to deliver sessions on relaxation and physical health. During the meeting the facilitators largely left facilitation to the external speakers who used a didactic style, which did not invite discussion or tailoring to the needs of group members. It was deemed by both coders that the facilitators could have coached external speakers on how to deliver a session in accordance with the Lifestyle Matters ethos and could have interjected during the sessions. An outcome from this session was to provide facilitators with further guidance on when and how to engage external speakers. This was delivered as an email to all facilitators ensuring they received consistent information.

A difference was also noted between the first and last waves at Site B where the assessed fidelity dropped from 87.5% to 70.8% but was still within the boundary set for satisfactory fidelity. Based on findings from the qualitative interviews, reasons for the reduction in scores could be explained by difficulties in time management to undertake planning and delivery of

For peer review only - http://bmjopen.bmj.com/site/about/guidelines.xhtml

multiple consecutive groups as well as the challenges presented by the dynamics within groups.

Group member performance

Excellent or very good fidelity was demonstrated for four out of the eight recorded groups (see Table 4). Only one group (Wave 1, Site A) was rated as being unsatisfactory (41%) with this being related to the same issue described earlier for 'Facilitator performance'.

Table 4: Fidelity scores for 'Group members'

	Group Member score /out of 39							
**Wave 1**	**(A) Group 1**				**(B) Group 1**			
	**Rater 1**	**Rater 2**	**Agreed score**	**%**	**Rater 1**	**Rater 2**	**Agreed score**	**%**
Video 1	34	33	33/39	**84.6%**	37	35	38/39	**97.4%**
Video 2	11	18	16/39	**41.0%**	30	29	29/39	**74.3%**
**Wave 3**	**(A) Group 6**				**(B) Group 5**			
	**Rater 1**	**Rater 2**	**Agreed score**	**%**	**Rater 1**	**Rater 2**	**Agreed score**	**%**
Video 1	33	31	33/39	**84.6%**	23	28	24/39	**61.5%**
Video 2	36	36	36/39	**92.3%**	26	26	26/39	**66.6%**

Excellent or very good fidelity was demonstrated for three out of eight identified domains with unsatisfactory evidence for only one; 'Confidence and hope'. However, it became evident that ability to score some criteria was affected by the nature of the activity's participants engaged in.

Inter-rater reliability

Kappa scores were calculated using linear weights[35].

For the assessment of training fidelity, the inter-rater reliability between coders was Kappa = 0.72 (p <.0.001), 95% CI (0.478, 0.964).

Inter-rater reliability for overall fidelity to intervention delivery across sites was Kappa = 0.63 (p <0.001), 95% CI (0.543, 0.719).

Inter-rater reliability for facilitator domains across both sites was Kappa = 0.55 (p <0.001), 95% CI (0.432, 0.678). For group members inter-rater reliability was Kappa = 0.72 (p <0.001), 95% CI (0.598, 0.844).

For peer review only - http://bmjopen.bmj.com/site/about/guidelines.xhtml

Qualitative findings

The following themes relevant to intervention fidelity were identified from analysis of the qualitative interviews conducted with facilitators following cessation of delivery. Other qualitative findings including those derived from interviews with participants are reported elsewhere[14 23].

Theme 1: Experience as an enabler of intervention delivery

Analysis of facilitator interviews revealed that they all considered that they had become more confident, skilled, and flexible over time. Also, that their commitment to the ethos of the programme was maintained:

> "*We've actually grown in confidence…it feels a very different place to where we were at the beginning... far more skilled... allowing for that flexibility and spontaneity" (Facilitator D).*

Theme 2: Views of how skills acquisition for intervention delivery could have been enhanced

Further training opportunities such as booster sessions or shadowing experienced facilitators were suggested to help consolidate knowledge of the intervention and improve fidelity further.

> *"…it would have been useful just to have…a follow up session just to see what kinds of things were coming up for people and have that, that kind of discussion" (Facilitator B).*

Regular self-review, using a group observation checklist was proposed by facilitators as a tool to enable them to reflect on delivery style and decision-making processes. (Facilitators were aware of the checklist as part of the fidelity work but did not have sight of the checklist).

> *"it may be more useful in future, if the facilitators could peer review themselves every three weeks against the checklist…scoring themselves and detailing an action against those that need to be achieved" (Facilitator A).*

Theme 3: Needs for more training to support greater understanding of group dynamics

One facilitator suggested that additional content in the training on how to avoid dependency relationships when facilitating using concrete examples case studies and written guidance would have been helpful.

For peer review only

"…what might breed relationships of dependency, what are the little things we [facilitators] don't realise we're doing, I think that might be something that could be added to the training" (Facilitator A)

The same facilitator thought that fostering and enabling independence was also important for the intervention and more time dedicated in the training was needed.

"…to process and assimilate all that…" (Facilitator A).

Another facilitator suggested that training should incorporate more on managing group dynamics and handling difficult people, and how to adapt sessions spontaneously when needed.

"…few trouble-shooting scenarios…" (Facilitator D).

Theme 4: Tailoring intervention components

One facilitator described modifying their approach to goal setting within the individual sessions where they felt participants had found the concept too formal or difficult to understand. Rather than talking about conscious goals they would integrate it subtly into the intervention.

"Interestingly you'd be approaching that same subject in a very subtle way over the weeks and weaving it into natural conversation, that was received a lot better…" (Facilitator 1).

Theme 5: Anxiety as a consequence of researcher observations

Two facilitators reported levels of performance anxiety due to the session being recorded, which they felt may have hindered their delivery of the intervention.

"I think it is fair to say that being recorded has significantly affected the performance of the facilitators due to nerves of being recorded. As such I don't think the recordings reflect what has actually taken place within the groups over time. We tick many of the boxes on the checklist, but the recordings I feel won't pick a lot of this up" (Facilitator D).

Discussion

The framework

The fidelity assessment framework designed for this study provided a satisfactory structure for monitoring and evaluating adherence to the intervention over time. We were able to

For peer review only

demonstrate good fidelity to training as well as delivery of the group component of the intervention but were unable to observe all intervention components including the individual sessions and the out of venue activities. It was significant, however, that participant adherence to the individual sessions was poor (32% of available sessions). As these sessions were deemed to be an essential component of the overall intervention, the reasons require further exploration. We were aware from the qualitative interview data that goal setting as part of the individual sessions, proved problematic for facilitators to deliver. This was attributed to the significant tailoring needed to meet the needs of individual participants.

There were however several shortcomings to the framework approach[29] for this type of intervention. Assessment frameworks are designed to rate set criteria, which by definition should be the same for all observations. This is not necessarily true of complex interventions such as Lifestyle Matters that have tailoring as an underlying mechanism. To accommodate this, the underpinning ethos of the intervention along with the manual and training content provided the basis of the observation checklists[39]. This allowed for a range of observed behaviours to be considered in relation to set criteria instead of only one behaviour signifying presence.

Fidelity assessment methodology and adaptation

We recognised the adaptive nature of the intervention and the resultant complexity for fidelity assessment at the time of delivering this study. Current debate is indeed questioning whether fidelity and adaptation can co-exist[40]. Being able to determine the difference between intervention ineffectiveness or alternatively failure to deliver as intended can significantly impact the implementation outcome[41]. This intervention includes a number of interlinked adaptations; namely selection of different components from a menu of possibilities, the context, the characteristics and needs of the participant and the group they are a member of and the characteristics and abilities of facilitators. Whether it is possible to measure a range of adaptions for such tailored interventions, and how this would be achieved requires further consideration.

Coding

Coding performance can raise concerns. Expectations of both the facilitators and those rating performances must be considered and moderated. All those involved should try to avoid pre-occupation with fidelity assessment at the cost of intervention delivery[42]. Coders were not

For peer review only

independent of the study, having attended the training as participant observers. Having the same knowledge and learning experience as those expected to deliver the intervention could enhance coder observations through greater understanding of observed behaviours and processes. Alternatively, using independent coders would reduce bias of unknown factors arising from personal knowledge of facilitators and increase the rigour of fidelity assessment. Overall we deduce that when the intervention is complex, ability to interpret what is occurring is required if accurate observations are to be recorded[43]. Using researchers with knowledge or experience of the intervention as coders may therefore be preferable.

Training and competence

One of the main challenges to fidelity was the delivery of high-quality in-depth training for a complex psycho-social intervention in just two days to a range of staff. Chances of variation in intervention delivery were deemed to lie in facilitator background and experience which are related to fidelity[6]. Evidence from the qualitative interviews also indicated that coverage of several core principles of the intervention including managing individual and group dynamics could have been improved during training. In this trial it was not possible for facilitators to give feedback on the training they had received but in real world implementation we would recommend including this. To scaffold fidelity we employed senior members of staff with experience working with older people and behaviour change interventions to supervise facilitators in the delivery of the intervention[44]. Due to the novelty of the intervention, it was not possible to identify staff with existing experience of delivering it or anything similar. Therefore, a recommendation for future research would be to include evaluation of fidelity to supervision in more detail including competence and content. This has since been explored in another trial, Journeying through Dementia[8].

The Lifestyle Matters manual and training are founded in 'occupational therapy'theory and practice and the facilitators were supervised by trained Occupational Therapists. However, in pursuance of evidence that the intervention might be delivered by a range of staff, the recruited facilitators came from a variety of professional backgrounds including social work and mental health advocacy as well occupational therapy. Further customisation of training may be required to meet the needs of a wider range of health and social care staff.

Some deterioration of skills, or drift, is common post training[25]. Although no additional training or booster sessions were provided during this study, facilitators received regular

For peer review only

supervision and occasionally received emailed ‘study updates’. These communications (approved by the Trial Management Group) clarified details on intervention delivery to help reduce potential facilitator drift. Any emergent ethical concerns would have been immediately acted on if identified during the fidelity assessment. Although fidelity to group intervention delivery was generally found to be good, further training opportunities such as booster sessions or shadowing experienced facilitators might be incorporated in future studies to test whether additional training consolidates knowledge of the intervention and further improves fidelity. In addition the group observation checklist used to assess adherence and competence[13 45] to deliver the intervention could be used by facilitators as a tool to self-regulate and thereby enhance fidelity[46].

Facilitators and supervisors

It has been suggested that due to the key role of facilitators they should be cognisant of the impact of fidelity[6]. The importance and nature of the planned fidelity assessment was explained to the facilitators during the two-day preparatory training before they took part in the assessment. This was beneficial in providing reassurance about the process including the observations.

Even though facilitators were from a range of backgrounds and had different experiences of working with older people in the community, this did not appear to negatively impact on intervention delivery[34]. The facilitator job description and person specification provided a priori level of credentials required to provide some consistency across facilitators. However, the previous point about responsiveness to needs for training and support by those running the study should be maintained. This also applies to supervisors who are not experienced in delivering the programme.

A ‘Good fit’ between providers and participants can be key to successful intervention delivery[25]. All facilitators in this trial were female and younger than the youngest participant but this did not appear to impact on delivery. The supervisors were generally older and more experienced in delivering occupational therapy-based interventions, which could be drawn upon during supervision sessions.

For peer review only - http://bmjopen.bmj.com/site/about/guidelines.xhtml

Receipt of intervention

Fidelity to the intervention increased between waves for Site A, but gradually decreased for Site B. This could have been due to the dynamics of the groups that were recorded; challenges with the methods used to assess fidelity or may have been indicative of facilitator drift over time. The only factor that could be identified to explain facilitator drift at site B was the time pressure they expressed in trying to prepare and deliver the group sessions as well as trying to organise and conduct individual sessions. This explanation is commensurate with the slight decline seen in 'Facilitator performance' and suggests that additional training may have been beneficial.

Fidelity was also influenced by receipt of the intervention. For example, we expected to observe group and individual goal setting opportunities in intervention sessions. However, from the qualitative interviews it was evident that the majority of participants struggled to engage with the concept of 'goal setting' and facilitators were required to make significant changes to their own understanding and how they presented the concept to participants[14]. Low fidelity therefore may not necessarily be associated with poor fidelity to delivery but may highlight a problem with the original intervention requirements, the nature of associated training and supervision, and subsequent criteria identified to evidence fidelity. Detailed analysis of feasibility study findings is therefore essential to establish clear baselines for behavioural interventions. Good design and piloting of tools is also important.

Practicalities and ethical considerations

The assessment only involved the video recording of a small number of weekly meetings out of the total provided and therefore it is inevitable that some facilitator and participant behaviours that may have affected intervention delivery were not observed. The assessment process also did not include observations of facilitators arranging and conducting individual sessions or out of venue activities which would have enhanced our understanding of the underlying intervention mechanisms. As previously described there were ethical considerations around participant confidentiality when assessing fidelity in a community setting[47]. Consideration should therefore be given to how fidelity assessment can be undertaken within any given setting without interfering with the intervention[48]. How many, when and what type of sessions to record should be dependent on the complexity of the trial and availability of resources. For example, it may be prudent to monitor consecutive sessions to capture more information regarding participant receipt and enactment. Behaviour change

For peer review only - http://bmjopen.bmj.com/site/about/guidelines.xhtml

may be more evident if an activity undertaken during one session can then be followed through to the next.

Psychosocial interventions are not designed to be passive; participants and facilitators bring their life experience, beliefs and expectations[34 49]. When determining intervention fidelity it is therefore important to consider how group dynamics and also environmental factors[50] impact not only on group composition but also group behaviour, especially when external factors to the intervention may be outside the control of those facilitating[49].

The findings obtained from assessment of intervention fidelity within this RCT were important, given the neutral results obtained from analysis of outcome data[10]. The fidelity assessment enabled identification of the aspects of intervention delivery that were delivered as per protocol as well as those aspects that were compromised such as delivery of the individual sessions and specifically ability to facilitate goal setting with participants. These issues help inform our understanding and interpretation of the trial results as well as indicating the challenges that future implementation would involve.

Conclusions

The outcomes from this fidelity assessment add to the growing literature on the use of fidelity assessment in trials and other studies of complex interventions. Psychosocial interventions that are flexible and offer a tailored approach in delivery may make fidelity assessment difficult, but not impossible. Paradoxically the importance of undertaking thorough fidelity assessment is enhanced for complex interventions, which have a number of components. Despite the challenges, this study has demonstrated that by testing methods identified in a replicable framework, fidelity assessment can be carried out successfully.

Further research should explore the potential of different observational methods for monitoring fidelity in psycho-social community based complex trials and the impact of these methods on intervention delivery.

Abbreviations

Behaviour Change Consortium (BCC)

Randomized controlled trial (RCT)

For peer review only - http://bmjopen.bmj.com/site/about/guidelines.xhtml

Declarations

Ethics approval and consent to participate

The study was approved by the South Yorkshire Research Ethics Committee 12/YH/0101, the National Institute for Social Care and Health Research (NISCHR) Permissions Co-ordinating Unit in Wales, Betsi Cadwaladr University Health Board. Written informed consent was obtained from all participants.

Consent for publication

We obtained written informed consent for the participants who took part in the interviews via the Trial Consent Form under the following statement:

"I understand and give permission for group meetings or interviews that I may be asked to take part to be video/audio recorded and for the resulting data to be used in reports and publications including direct quotation."

This information is held as part of the archived record of the trial. Only anonymised non-identifiable data are used in this report as per written consent.

Availability of data and material

Data are available upon reasonable request. The datasets generated and analysed for this study will be available upon request from the authors.

Competing interests

GM and CC are the original authors of the published Lifestyle Matters manualised programme. No other authors have any competing interests.

Funding

The trial was funded through the Medical Research Council (MRC) Lifelong Health and Wellbeing (LLHW) programme, grant number G1001406, ISRCTN is 67209155. The views and opinions expressed in this paper are those of the authors and do not necessarily reflect those of the MRC. Primary Care Research Network (PCRN) funding was accessed to support recruitment activity in GP surgeries in Sheffield and NISCHR provided support in North Wales. The funder has reviewed and approved the trial design including methods of data collection, analysis and interpretation of data.

For peer review only - http://bmjopen.bmj.com/site/about/guidelines.xhtml

Author contributions

GM and CC obtained the funding. GM, KS and CC developed the research design, fidelity assessment framework and data collection tools. KS managed the research and undertook data collection. KS and GM analysed and interpreted the data. KS drafted the manuscript. All authors critically reviewed and approved the manuscript.

Acknowledgements

PPI contributors. Prof John Brazier, Dr Daniel Hind, Prof Stephen Walters, Dr Gill Windle, Prof Robert Woods (TMG) advised on the trial protocol including the process evaluation and fidelity assessment. Dr Pip Logan, Dr Jennifer Wenborn, Dr Linda Sheppard, Dr Fiona Goudie of the Trial Steering Committee (TSC) and Dr Mona Kanaan, Prof Avril Drummond and Dr Claire Ballinger of the Data Monitoring and Ethics Committee (DMEC) advised on and critically reviewed the trial protocol including the process evaluation and fidelity assessment.

References

1. McDermott O, Charlesworth G, Hogervorst E, et al. Psychosocial interventions for people with dementia: a synthesis of systematic reviews. *Aging & mental health* 2019;23(4):393-403. doi: 10.1080/13607863.2017.1423031 [published Online First: 2018/01/18]

2. Moniz-Cook E, Vernooij-Dassen M, Woods B, et al. Psychosocial interventions in dementia care research: the INTERDEM manifesto. *Aging & mental health* 2011;15(3):283-90. doi: 10.1080/13607863.2010.543665 [published Online First: 2011/04/15]

3. National Institute for Health and Care Excellence. Public health guideline [PH16]. Mental wellbeing in over 65s: occupational therapy and physical activity interventions 2008 [Available from: https://www.nice.org.uk/guidance/ph16 accessed 03Januray2018.

4. Government Office for Science. Foresight Mental Capital and Wellbeing: Making the most of ourselves in the 21st century 2008 [Available from: http://www.bis.gov.uk/assets/biscore/corporate/migratedD/ec_group/116-08-FO_b accessed January 2013.

For peer review only

5. Toomey E, Hardeman W, Hankonen N, et al. Focusing on fidelity: narrative review and recommendations for improving intervention fidelity within trials of health behaviour change interventions. *Health Psychology and Behavioral Medicine* 2020;8(1):132-51. doi: 10.1080/21642850.2020.1738935

6. Spillane V, Byrne M, Byrne M, et al. Monitoring treatment fidelity in a randomised controlled trial of a complex intervention. *J Adv Nurs* 2007;60(3):343-52.

7. O'Malley KA, Qualls SH. Application of treatment fidelity in tailored caregiver interventions. *Aging & mental health* 2020;24(12):2094-102. doi: 10.1080/13607863.2019.1647134

8. Sprange K, Beresford-Dent J, Mountain G, et al. Assessing fidelity of a community based psychosocial intervention for people with mild dementia within a large randomised controlled trial. *BMC Geriatrics* 2021;21(1):119. doi: 10.1186/s12877-021-02070-8

9. Walton H, Tombor I, Burgess J, et al. Measuring fidelity of delivery of the Community Occupational Therapy in Dementia-UK intervention. *BMC Geriatrics* 2019;19(1):364. doi: 10.1186/s12877-019-1385-7

10. Mountain G, Windle G, Hind D, et al. A preventative lifestyle intervention for older adults (lifestyle matters): a randomised controlled trial. *Age and Ageing* 2017;46(4):627-34. doi: 10.1093/ageing/afx021

11. Clark F, Azen S, Zemke R, et al. Occupational Therapy for Independent-Living Older Adults. *Journal of the American Medical Association* 1997;278(16):1321-6.

12. Clark F, Jackson J, Carlson M, et al. Effectiveness of a lifestyle intervention in promoting the well-being of independently living older people: results of the Well Elderly 2 Randomised Controlled Trial. *Journal of Epidemiology & Community Health* 2011

13. Moncher F, Prinz R. Treatment fidelity in outcome studies. *Clinical Psychology Review* 1991;11:247-66.

14. Mountain G, Sprange K, Chatters R. Lifestyle Matters Randomized Controlled Trial of a Preventive Health Intervention for Older People: Qualitative Sub Study with Participants and Intervention Facilitators. *Clinical interventions in aging* 2020;15:239-53. doi: 10.2147/cia.s232108 [published Online First: 2020/02/29]

15. Medical Research Council. Developing and evaluating complex interventions [Available from: https://mrc.ukri.org/documents/pdf/complex-interventions-guidance/ accessed 18Mar2021.

For peer review only

16. Bandura. A. Self-efficacy: The exercise of control.: New York: Freeman 1997.

17. Luszczynska A, Gutierrez-Dona B, Schwarzer R. General self-efficacy in various domains of human functioning: Evidence from five countries. *International Journal of Psychology* 2005;40(2):80-89.

18. Bandura A. Self-efficacy mechanism in human agency. *American Psychologist* 1982;37(2):122-47. doi: 10.1037/0003-066X.37.2.122

19. Rowe J, Kahn R. Successful Ageing: New York: Pantheon 1989.

20. Hasson H, Blomberg S, Duner A. Fidelity and moderating factors in complex interventions: a case study of a continuum of care program for frail elderly people in health and social care. *Implement Sci* 2012;7:23. doi: 10.1186/1748-5908-7-23 [published Online First: 2012/03/23]

21. Walton H, Spector A, Tombor I, et al. Measures of fidelity of delivery of, and engagement with, complex, face-to-face health behaviour change interventions: A systematic review of measure quality. *British journal of health psychology* 2017;22(4):872-903. doi: 10.1111/bjhp.12260 [published Online First: 2017/08/02]

22. Brooke P, Bullock R. Validation of a 6 item cognitive impairment test with a view to primary care usage. *International journal of geriatric psychiatry* 1999;14:936-40.

23. Chatters R, Roberts J, Mountain G, et al. The long-term (24-month) effect on health and well-being of the Lifestyle Matters community-based intervention in people aged 65 years and over: a qualitative study. *BMJ open* 2017;7(9):e016711. doi: 10.1136/bmjopen-2017-016711

24. Sprange K, Mountain GA, Brazier J, et al. Lifestyle Matters for maintenance of health and wellbeing in people aged 65 years and over: study protocol for a randomised controlled trial. *Trials* 2013;14:302-02. doi: 10.1186/1745-6215-14-302

25. Borrelli B. The assessment, monitoring, and enhancement of treatment fidelity in public health clinical trials. *Journal of Public Health Dentistry* 2011;71:S52-S63.

26. Carroll C, Patterson M, Wood S, et al. A conceptual framework for implementation fidelity. *Implement Sci* 2007;2:40. doi: 10.1186/1748-5908-2-40 [published Online First: 2007/12/07]

27. Craig C, Mountain G. Lifestyle Matters: an occpational approach to healthy ageing: Bicester: Speechmark 2007.

28. Hawe P, Shiell A, Riley T. Complex interventions: how “out of control” can a randomised controlled trial be? *BMJ* 2004;328(7455):1561-63. doi: 10.1136/bmj.328.7455.1561

For peer review only - http://bmjopen.bmj.com/site/about/guidelines.xhtml

29. Bellg A, Resnick B, Sharp Minicucci D, et al. Enhancing treatment fidelity in health behaviour change studies: Best practices and recommendations from the NIH Behaviour Change Consortium. *Health Psychology* 2004;23(5):443-51.

30. National Institute for Health and Clinical Excellence (NICE). Public Guidance 6. Behaviour change: general approaches 2007 [Available from: http://guidance.nice.org.uk/PH6/Guidance/pdf/English.

31. Mountain G, Mozley C, Craig C, et al. Occupational therapy led health promotion for older people: feasibility of the lifestyle matters programme. *British Journal of Occupational Therapy* 2008;71(10):406-13.

32. Pope C, Mays N. Reaching the parts other methods cannot reach: an introduction to qualitative methods in health and health services research. *BMJ : British Medical Journal* 1995;311(6996):42-45.

33. Spradley JP. Participant observation: Waveland Press 2016.

34. Masterson-Algar P, Burton CR, Rycroft-Malone J, et al. Towards a programme theory for fidelity in the evaluation of complex interventions. *J Eval Clin Pract* 2014;20(4):445-52. doi: 10.1111/jep.12174 [published Online First: 2014/05/21]

35. McHugh ML. Interrater reliability: the kappa statistic. *Biochem Med (Zagreb)* 2012;22(3):276-82. [published Online First: 2012/10/25]

36. Braun V, Clarke V. To saturate or not to saturate? Questioning data saturation as a useful concept for thematic analysis and sample-size rationales. *Qualitative Research in Sport, Exercise and Health* 2021;13(2):201-16. doi: 10.1080/2159676X.2019.1704846

37. Gale N, Heath G, Cameron E, et al. Using the framework method for the analysis of qualitative data in multi-disciplinary health research. *BMC Med Res Methodol* 2013;13(117)

38. Ritchie J, Spencer L. Qualitative data analysis for applied policy research: The Qualitative Researcher's Companion: SAGE Publications 2002.

39. Mars T, Ellard D, Carnes D, et al. Fidelity in complex behaviour change interventions: a standardised approach to evaluate intervention integrity. *BMJ open* 2013;3(11):e003555. doi: 10.1136/bmjopen-2013-003555 [published Online First: 2013/11/19]

For peer review only - http://bmjopen.bmj.com/site/about/guidelines.xhtml

40. Pérez D, Van der Stuyft P, Zabala MdC, et al. A modified theoretical framework to assess implementation fidelity of adaptive public health interventions. *Implementation Science* 2016;11(1):91. doi: 10.1186/s13012-016-0457-8

41. Proctor E, Silmere H, Raghavan R, et al. Outcomes for implementation research: conceptual distinctions, measurement challenges, and research agenda. *Administration and policy in mental health* 2011;38(2):65-76. doi: 10.1007/s10488-010-0319-7 [published Online First: 2010/10/20]

42. Dumas J, Lynch A, Laughlin J, et al. Promoting Intervention Fidelity. Conceptual Issues, Methods, and Preliminary Results from the EARLY ALLIANCE Prevention Trial. *American Journal of Preventive Medicine* 2001;20(1S)

43. Duncan EAS, Nicol MM. Subtle Realism and Occupational Therapy: An Alternative Approach to Knowledge Generation and Evaluation. *The British Journal of Occupational Therapy* 2004;67(10):453-56. doi: 10.1177/030802260406701006

44. Beck C, McSweeney JC, Richards KC, et al. Challenges in tailored intervention research. *Nursing outlook* 2010;58(2):104-10. doi: 10.1016/j.outlook.2009.10.004 [published Online First: 2010/04/07]

45. Carroll KM, Nich C, Sifry RL, et al. A general system for evaluating therapist adherence and competence in psychotherapy research in the addictions. *Drug and alcohol dependence* 2000;57(3):225-38. doi: 10.1016/s0376-8716(99)00049-6 [published Online First: 2000/02/08]

46. Song M, Happ M, Sandelowski M. Development of a tool to assess fidelity to a psycho-educational intervention. *J Adv Nurs* 2010;66(3):673-82.

47. Watts JH. Ethical and practical challenges of participant observation in sensitive health research. *International Journal of Social Research Methodology* 2011;14(4):301-12. doi: 10.1080/13645579.2010.517658

48. Resnick B, Inguito P, Orwig D, et al. Treatment Fidelity in Behaviour Change Research: a case example. *Nursing Research* 2005;54(2):139-43.

49. Moore G, Audrey S, Barker M, et al. Process evaluation in complex public health intervention studies: the need for guidance. *J Epidemiol Community Health* 2014;68(2):101-2. doi: 10.1136/jech-2013-202869 [published Online First: 2013/09/12]

For peer review only

50. Hoddinott P, Allan K, Avenell A, et al. Group interventions to improve health outcomes: a framework for their design and delivery. *BMC Public Health* 2010;10(800)

For peer review only

Lifestyle Matters Additional File 1

Contents

Document 1: Lifestyle Matters training delivery observation checklist 2

Document 2: Lifestyle Matters intervention delivery video checklist 3

For peer review only

Document 1: Lifestyle Matters training delivery observation checklist

TRAINER	
**Domain**	**Criteria**
Trainer	1. The trainer is the same for all training
	2. The trainer is an author of the programme
	3. Demonstrates a range of group work skills (didactic teaching, peer sharing, interactive activities, active experimentation etc)
Materials	4. PowerPoint presentations and materials directly relate to the goals of the training
	5. Provides a CD of training content and materials to all trainees
Tailoring	6. Training is tailored to recipients' skill level, experience or work background
	7. Training is tailored in response to the observed needs of the trainees, and feedback from the trainees during the sessions
Content	8. The goals of the training are covered
	9. Examples of the core themes and examples of subsidiary themes are covered
	10. Mandatory themes are covered:<br>• A celebration of achievements<br>• Activity and health<br>• Endings
**TRAINEES**	(Performance in discussion, role play, problem solving, expressive exercises, reflective exercises shared, written tests)
**Domain**	**Criteria**
Understanding	11. Critically discuss demographic changes and the concept of active ageing
	12. Discuss the evidence for Lifestyle matters
	13. Identify the ideas that are key to Lifestyle Matters, including<br>• enabling group members to do things for themselves<br>• transferring skills from the safety of the group to their wider lives
	14. Discuss the content of the Lifestyle Matters manual and how to apply it
Self -awareness	15. Share their prior expertise, hopes and expectations of the training
	16. Share their own perceptions of ageing and how this may impact on the programme
	17. Reflect on and share their own facilitation style and skills
Skill acquisition	18. Use the LifeStyle Matters manual to practice planning sessions within themes
	19. Identify how to locate and access community resources
	20. Respond to potential scenarios, using creative problem solving and considering individual needs (including risk taking)
Group work	21. Understand and demonstrate a range of group work skills (didactic teaching, peer sharing, interactive activities, active experimentation etc)
	22. Understand and demonstrate how to manage group dynamics
	23. Understand and demonstrate an enabling style of group facilitation
	24. Demonstrate how to tailor the intervention to the needs and choices of group members
Individual sessions	25. Explore the value of 1:1 sessions and potential activities to use in these
Group outings	26. Explore the value or outings and how to help group members to plan them
Reflection & evaluation	27. Explore reflective practice and discuss its value
	28. Practice evaluating and planning actions
Supervision	29. Discuss the value and principles of supervision

For peer review only

Document 2: Lifestyle Matters intervention delivery video checklist

FACILITATOR	
**Domain**	**Criteria**
Resources	30. Two facilitators deliver
Personal qualities	31. Shows warmth and empathy
	32. Listens actively and respectfully
Enabling	33. builds on the assets, skills and abilities that each person brings
	34. Encourages the group to make choices
Group work skills	35. Facilitates communication and interaction (to ground rules?)
	36. Draws in quiet members of the group
	37. Facilitates peer exchange and guidance, and peer modelling
	38. Manages space, time and opportunity effectively
	39. Facilitates the members to explore differences between them, or conflict and resolution
	40. Builds mutual support and group cohesion
Goals and needs	41. Balances meeting individual needs and goals and group needs
	42. Balances sufficient challenge in activities with ensuring they are achievable
	43. Tailors activities to the needs of members
Content	44. Focuses on the themes in the programme, or new themes chosen by group members
	45. Uses group activities, not just discussion (activities from the manual or from elsewhere)
	46. With members, selects and plans group outings that explore and extend the themes in the programme
**GROUP MEMBERS**	
**Domain**	**Criteria**
Understanding	47. The relationship between activity, health and aging, or one of the other themes from the programme
Occupational self-analysis	48. Analyse their own daily occupations (their significance, impact on health, components) and make choices
	49. Sharing and reflecting on experiences of participating in activities (Occupational storytelling)
Goal setting	50. Own goals identified within the group and within activities
Grading and layering	51. Grading and layering of activities to constantly revisit and extend skills and build confidence
Mutual support	52. Show mutual appreciation and support
Creativity	53. Practice any of: self-expression, innovation, problem solving and adaptation within the safety of the group
	54. Engage in enjoyable and stimulating activities
Older people as experts	55. Acknowledge and appreciate their own skills and how they can contribute to the group
	56. Bring in and share their own skills and knowledge
	57. Group members in turn take leadership or contribute ideas
Transfer to everyday life	58. build skills, solutions or confidence within the group
	59. Report the transferring of skills, solutions or confidence from the group to wider life
Change over time	60. Plans for individuals are taken forward, whilst respecting each person’s need to balance opportunities and risk
Confidence and hope	61. Achievements are celebrated
	62. Adventures and dreams are welcomed

For peer review only
